# Synthetic Heterocyclic Derivatives as Kinase Inhibitors Tested for the Treatment of Neuroblastoma †

**DOI:** 10.3390/molecules26237069

**Published:** 2021-11-23

**Authors:** Francesca Musumeci, Annarita Cianciusi, Ilaria D’Agostino, Giancarlo Grossi, Anna Carbone, Silvia Schenone

**Affiliations:** 1 Department of Pharmacy, University of Genoa, Viale Benedetto XV, 3, 16132 Genoa, Italy; francesca.musumeci@unige.it (F.M.); annarita.cianciusi@edu.unige.it (A.C.); grossi@difar.unige.it (G.G.); carbone@difar.unige.it (A.C.); 2 Department of Biotechnology, Chemistry and Pharmacy, University of Siena, Via Aldo Moro 2, 53100 Siena, Italy; ilaria.dagostino91@gmail.com

**Keywords:** neuroblastoma, kinase inhibitors, extracranial tumor, pediatric cancers, clinical trials

## Abstract

In the last few years, small molecules endowed with different heterocyclic scaffolds have been developed as kinase inhibitors. Some of them are being tested at preclinical or clinical levels for the potential treatment of neuroblastoma (NB). This disease is the most common extracranial solid tumor in childhood and is responsible for 10% to 15% of pediatric cancer deaths. Despite the availability of some treatments, including the use of very toxic cytotoxic chemotherapeutic agents, high-risk (HR)-NB patients still have a poor prognosis and a survival rate below 50%. For these reasons, new pharmacological options are urgently needed. This review focuses on synthetic heterocyclic compounds published in the last five years, which showed at least some activity on this severe disease and act as kinase inhibitors. The specific mechanism of action, selectivity, and biological activity of these drug candidates are described, when established. Moreover, the most remarkable clinical trials are reported. Importantly, kinase inhibitors approved for other diseases have shown to be active and endowed with lower toxicity compared to conventional cytotoxic agents. The data collected in this article can be particularly useful for the researchers working in this area.

## 1. Introduction

Neuroblastoma (NB) is the most common extracranial solid tumor in childhood and is responsible for 10% to 15% of pediatric cancer deaths [1]. This embryonic tumor originates from neural crest-related precursor cells [2], a multipotent and migratory cell population that gives rise to diverse cell lineages, including Schwann cells, melanocytes, craniofacial cartilage, bone and smooth muscle cells, peripheral and enteric neurons, and glia. Under normal conditions, neural crest cell precursors migrate from the dorsal neural tube and differentiate upon reaching their appropriate locations into tissues and organs of the sympathetic nervous system. Pathological defects in neural crest cell migration, maturation, or differentiation can lead to the development of NB [3].

NB has high genetic, biological, clinical, and morphological heterogeneity, which made this tumor very difficult to treat [4]. Moreover, this heterogeneity leads to a highly variable prognosis, ranging from spontaneous regression to widespread metastatic disease that is unresponsive to treatment. Consequently, research efforts have focused on the identification of novel biomarkers for stratification and prognostication as well as novel cellular pathways that can be targeted by new treatment strategies [3].

Risk stratification and prognosis for NB is an ongoing area of scientific research. The most recent NB classification system was developed by the International Neuroblastoma Risk Group (INRG) and utilizes seven clinical and biological variables [5]. Within the INRG system, the presence of MYCN oncogene amplification is a key determinant to designate NB as at high-risk (HR) [6].

A bulk of literature is focused on the involvement of this oncogene in NB, particularly in the evaluation of potential drugs in MYCN-expressing NB. In fact, an amplification of the MYCN locus, which is located on chromosome 2p24, is present in approximately 20% of NB patients [7], and the degree of amplification is associated with advanced disease, unfavorable biologic features, and poor outcomes. MYCN encodes the transcriptional factor N-Myc, a member of the Myc family of proto-oncogenes that includes other members such as c-Myc and L-Myc. The MYC family proteins function as transcriptional regulators and are involved with cell differentiation, growth, and proliferation. Distinctly from ubiquitously expressed c-Myc, is N-Myc expression is mainly restricted to the nervous system and mesenchymal tissues during particular embryonal stages and is present at very low levels in adult tissues. N-Myc also plays an essential role in normal brain development. Moreover, other studies have revealed the presence of several genomic alterations in NB beyond MYCN amplification, such as mutations of anaplastic lymphoma kinase (ALK), and copy number aberrations of chromosomes [7].

Despite the availability of some treatments, HR-NB patients still have a poor prognosis and a survival rate below 50% [8]. The standard NB treatments used include surgery, chemotherapy, radiotherapy, and biotherapy. When feasible, the initial surgery is attempted, especially for the control of localized tumors with favorable biological features in low-risk patients [9]. By contrast, the treatment of patients with localized tumors with unfavorable biological features, especially MYCN amplification, remains controversial. This diagnostic scenario has a rapid progression and dissemination with surgery alone and is allocated to the HR protocol and a multimodal therapy is required. This treatment generally consists of intensive induction therapy to induce tumor remission, followed by a myeloablative consolidation regimen to eradicate any residual cancer cells. Most induction regimens utilize cytotoxic agents, including platinum derivatives, cyclophosphamide, ifosfamide, doxorubicin, etoposide, irinotecan, and topotecan [9].

Recently, the novel FDA-approved drug verteporfin (VP) has been used to manage NB progression and patient relapse [10]. VP is an inhibitor of the YAP/TAZ-TEAD complex [11] which is involved in NB drug resistance exhibited [12]. Furthermore, VP down-regulates CD133 protein encoded by *PROM1*, which is also expressed in human primary NB cells with aggressive phenotypes [13]. The effects of VP in combination with standard anti-NB chemotherapy were evaluated and showed that this drug improved etoposide and cisplatin activity.

Retinoids are active against NB both as differentiation inducers and cytotoxic agents [14]. A randomized phase III trial of 13-cis-retinoic acid following myeloablative chemotherapy established its importance in therapy for HR-patients especially in combination with ch14.18 antibody and cytokines. Notably, since retinoids can potentially counteract the efficacy of conventional chemotherapy, they are generally used separately in a maintenance phase of therapy. The synthetic retinoid Fenretinide has been investigated as a potential agent to treat NB and has been formulated as an intravenous lipid emulsion to reach a more manageable safety profile and higher plasma steady-state concentrations of the active metabolite [15].

Although these treatment options, NB recurrence makes the use of alternative approaches essential, i.e., the use of cytokines, antibodies, and other cellular therapies [16]. In this context, the most established immunotherapies for NB involve antibodies and antibody-based (e.g., immune cytokines) therapies directed against GD2, a ganglioside highly expressed on NB cells, whose role is thought to be important for the attachment of tumor cells to extracellular matrix proteins [17]. Several antibodies against GD2 have been developed for clinical use and, recently, a humanized anti-GD2 monoclonal antibody (hu3F8-IgG1), Naxitamab (DANYELZA^®^, naxitamab-gqgk), has received its first approval in the USA to treat NB in children at least 1-year-old and adults with relapsed HR-NB [18]. Dinutuximab (ch14.18) is a chimeric monoclonal antibody approved by FDA in 2015 for the treatment of HR-NB, in combination with 13-cis-retinoic acid (RA), interleukin-2 (IL-2), and granulocyte-macrophage colony-stimulating factor (GM-CSF) [19]. It is a second-line treatment, indicated only when at least a partial response to first-line multimodal therapy has occurred [20].

Protein kinases are one of the widest families of enzymes in mammalians (human genome, known as kinome, encodes for about 500 protein kinases) [21] and catalyze the transfer of the terminal phosphate group from nucleotide triphosphates, such as ATP, to tyrosine, serine, or threonine OH-groups present on their protein substrates, resulting in conformational and functional changes of such proteins. They are classified in serine-threonine kinases (STK), tyrosine kinases (TKs), and nonspecific TK based on the substrate amino acid that is phosphorylated. Most protein kinases are receptors and are located on the cell surface, while other kinases are intracellular (non-receptors) and have a role in cell signaling. The formers are activated by the binding with specific ligands and initiate intracellular pathways. This heterogeneous class of enzymes plays crucial roles in the growth, proliferation, differentiation, and apoptosis of healthy cells and, after mutations they can undergo unregulated expression, resulting in an alteration of all these cellular activities and the rise of tumor cells [21]. In fact, they are often overexpressed or hyperactivated in different types of cancers, including NB [22,23,24].

**Figure 1:**
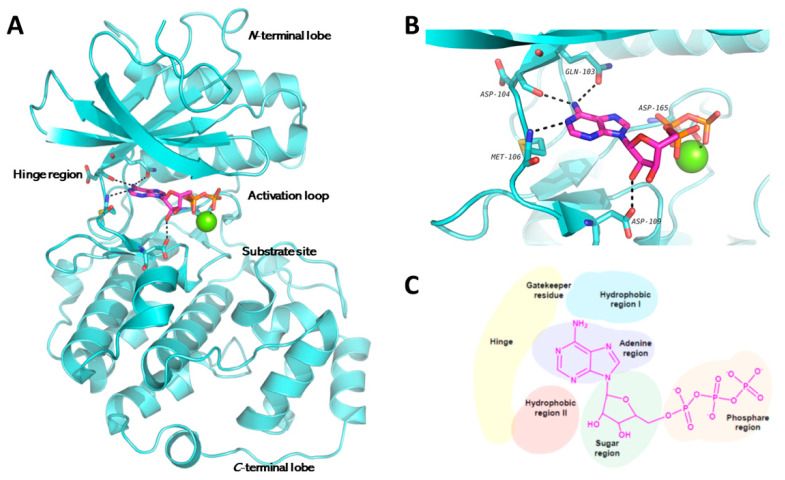
Structure and subregions of a typical protein kinase domain. (**A**) Structure of a typical protein kinase. The protein backbone is shown in cartoon form in cyan, the magnesium ion is shown as a green sphere, and ATP is in magenta sticks. From PDB structure 4GT3 [29] (ERK-2) as a representative protein kinase. (**B**) Zoom of ATP binding site. Hydrogen bonds with labeled amino acid residues and magnesium chelation are represented as black dashed lines. (**C**) Schematic representation of ATP binding site divided into subregions. Images A and B were prepared by PyMol Molecular Graphics System [30].

As regards a molecular insight into the ATP binding site (see Figure 1), kinases bind ATP molecules in a deep cleft located between the two lobes of the catalytic domain whose folding is due to highly conserved characteristic secondary structure elements of the protein. In particular, the ATP binding is stabilized by multiple hydrogen bonding patterns between the ATP heteroaromatic adenine ring and the hinge region (also known as adenine region) located between the two kinase lobes. Moreover, kinases share a conserved activation loop, marked by DFG and APE motifs [25,26]. Hence, the high degree of residues conservation [27] makes kinases very attractive pharmacological targets for anticancer, and, in particular, anti-NB treatment. In fact, the inhibition of the phosphorylation pathways represents a valuable strategy to search for new anticancer agents and several kinase inhibitors, such as the ATP-competitive types I (that bind the active protein conformation) and II (that preferentially bind the inactive one), and allosteric types III and IV inhibitors have been developed in recent years. The interest in these inhibitors is also due to their lower toxicity compared to the traditional chemotherapeutic cytotoxic agents, are orally administered, and frequently lead to exceptional results in the treatment of specific tumors [28]. However, to date, although several kinase inhibitors entered the market for the treatment of malignancies, no one has been still approved, alone or in combination, for the treatment of NB, but many of these compounds are being tested on this cancer.

In this review, we report the studies on kinase inhibitors tested in different models of NB, particularly focusing on the last five years. The inhibitors are described based on the targeted kinases. Furthermore, the currently active clinical trials involving kinase inhibitors, alone or in combination with other compounds, are reported. Moreover, for most compounds, the role of the heterocyclic core and the structural motifs in the interaction with the targeted protein(s) is briefly described.

## 2. STK Inhibitors

### 2.1. CDK Inhibitors

Cyclin-dependent kinases (CDKs) are a family of STKs which, in combination with the appropriate cyclin, regulate the progression through the cell cycle. Their deregulation has frequently been observed in cancer. In particular, CDK4 and CDK6 are hyperactivated in NB and correlated with poor prognosis [31].

CDK inhibitors were found to slow tumor growth and dissemination [32], by inhibiting cell proliferation and inducing apoptosis both in vitro and in vivo and relevant combinatorial effects with traditional chemotherapeutics have been observed [33]. Compounds endowed with polynitrogenated heterocycles, such as 2-aminopyrimidine, pyrrolo[2,3-*d*]pyrimidine, pyrrolo[2,3-*d*]pyrimidin-7-one, and pyrazolo[1,5-*a*]pyrimidine, fit the hinge region of the ATP binding site through several hydrogen bondings. These scaffolds are the most described in literature and exert very potent inhibitory activity both in enzymatic (nanomolar) and cellular (micromolar) assays.

#### 2.1.1. Roniciclib

**Figure 2:**
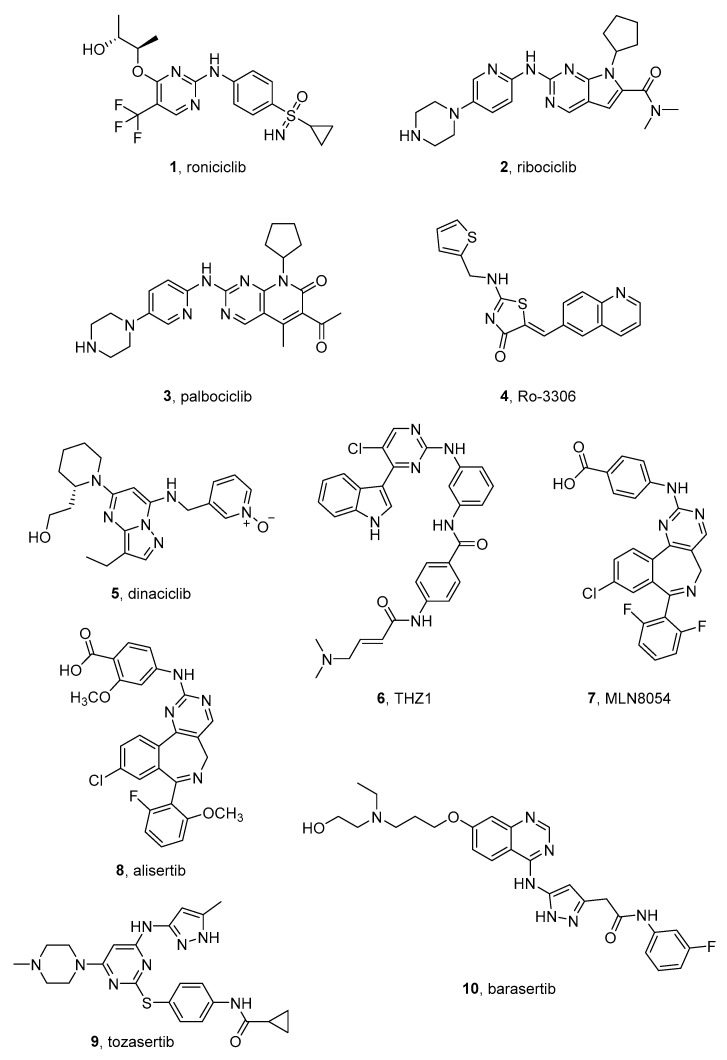
Structures of CDK and AURK inhibitors.

Roniciclib (BAY 1000394) (**1** in Figure 2), by Bayer, is a type I pan-CDK inhibitor, with IC_50_ values in the range 5–25 nM for CDK1, CDK2, CDK3, CDK4, CDK7, and CDK9. Moreover, it inhibits the expression of c-Myc and, consequently, the expression of the c-Myc-targeted cell cycle regulators CCND1, CDKN1A, and CDKN2D. This compound, which is endowed with a 2-aminopyrimidine core, binds the active conformation of CDKs and, for this reason, is classified among the type I kinase inhibitors [34]. It is being tested in phase I/II clinical trials for different malignancies, especially for lung cancer, in combination with cytotoxic agents [35].

Regarding NB, Yang et al. reported that the compound shows antiproliferative activity on MA and MN HR-NB cells by inducing apoptosis. Moreover, the authors investigated the role of c-Myc in HR-NB, by performing an in-depth analysis of the transcriptome, which showed a hyperactivated c-Myc downstream network. This result shows the involvement of the c-Myc gene in certain HR forms of NB and would be useful for the development of personalized treatments for patients expressing high levels of c-Myc [36]. Very recently, it has been reported that roniciclib is active also on NB stem cells, which are a population of undifferentiated cells leading to an unfavorable prognosis. Ognibene et al. demonstrated the anticancer effects of the compound on neurospheres derived from NB stem-cell-like and in an orthotopic NB mouse model [37].

A cocrystal of roniciclib bound to CDK2 (PDB code: 5IEV) [38] highlighted the key role of the aminopyrimidine scaffold, its trifluoromethyl group in C5, and the ether moiety in C4 for the potency in tumor growth inhibition in HeLa-MaTu xenograft models. Moreover, the binding causes a conformational adaption of CDK2 and a displacement of a specific loop residue in the proximity of the cyclin interface [34].

#### 2.1.2. Ribociclib

Ribociclib (LEE011) (**2** in Figure 2), bearing a pyrrolo[2,3-*d*]pyrimidine scaffold, is an orally bioavailable CDK4/6 inhibitor by Novartis and is active on different solid tumors [39]. Ribociclib possesses activity in the nanomolar range (1–10 nM) towards CDK4 [40] and is able to arrest NB growth in several cell lines with a mean IC_50_ of 306 ± 68 nM [41].

In 2017, this compound was approved by FDA and EMA for treatment of HR-positive, HER2-negative advanced, or metastatic breast cancers in combination with an aromatase inhibitor [42]. In the same year, a phase 1 study in pediatric patients with different solid tumors, including NB, evaluated the maximum tolerated dose, safety, and pharmacokinetics of the compound. The latter showed an acceptable safety profile and could be used in combination with other anticancer agents in the treatment of NB [43]. Ribociclib was tested in NB models in association with the mitogen-activated protein kinase (MEK) inhibitor binimetinib (see next paragraph). The rationale of this combination is based on the knowledge that relapsed NB can harbor a hyperactivated extracellular signal-regulated kinase (ERK) which is inhibited by MEK inhibitors. This treatment led to synergistic antiproliferative effects on different NB cell lines and three out of four xenograft models. These data suggested the use of ribociclib-binimetinib combination in relapsed HR-NB patients and, in particular, in patients with hyperactivated RAS-MAPK signaling [44]. Furthermore, ribociclib was associated with the ALK inhibitor ceritinib (see next paragraph). This combination showed a synergistic effect especially in NB cell lines with *ALK* mutations, where it led to enhanced growth inhibition, cell-cycle arrest, and caspase-independent cell death, in comparison with the two drugs used alone, and was also active in NB xenografts harboring *ALK*-F1174L and F1245C mutations [45].

A cocrystal CDK6 and ribociclib (PDB code: 5L2T) [46] showed the fitting of the 2-aminopyrimidine scaffold in the CDK6 hinge region with the positively-charged piperazine ring that lies against a solvent-exposed ridge. Moreover, the presence of the dimethylamino group seems to be essential for the selectivity towards CD4/CD6 kinases [47].

#### 2.1.3. Palbociclib

Palbociclib (**3** in Figure 2) is a pyrido[2,3-*d*]pyrimidin-7-one and is a type I CDK 4/6 selective inhibitor by Pfizer, approved in 2017 for the treatment of HR-positive and HER2-negative breast cancer. Palbociclib possesses IC_50_ values of 11 and 16 nM toward CDK4 and CDK6, respectively, [48] and reduces the cell growth in different NB cell lines. In particular, it showed IC_50_s of 261 and 672 nM toward IMR-32 and SH-SY5Y cell lines [49]. Kojima et al. tested the compound in association with RO-3306 (**4** in Figure 2), a quinolinyl thiazolinone compound that acts as a CDK1 inhibitor [50], in preclinical NB chick embryo models. This combination displays antiproliferative activity on SK-N-AS and BE(2)C cell lines engrafted in chick embryos. Moreover, Swadi et al. observed a reduction in the expression of genes involved in metastasis formation [32].

Palbociclib was cocrystallized with CDK6 (PDB code: 5L2I) [51] and, in addition to data collected for ribociclib, the X-ray analysis shows that the methylketone and methyl groups on the heterocyclic core could be crucial for the CDK4/6 selectivity. In fact, they are larger substituents that cannot accommodate the ATP-binding site belonging to other kinases [47].

#### 2.1.4. Dinaciclib

Dinaciclib (SCH-727965, MK-7965) (**5** in Figure 2) is a type I CDK inhibitor by Merck containing a pyrazolo[1,5-*a*]pyrimidine scaffold. The compound was granted orphan drug status by the FDA in 2011 and, currently, is being tested in clinical trials for different malignancies. It exerts an antiproliferative activity on a broad panel of NB cell lines, inhibiting CDK2 and CDK9 and increasing the activity of cytotoxic agents, such as doxorubicin and etoposide. Moreover, dinaciclib shows anticancer efficacy in orthotopic xenograft mouse models derived from two NB cell lines and markedly decreases tumor development in the TH-MYCN transgenic NB mouse model [33].

Two recent studies by the same research group evaluated the combination of dinaciclib with AZD515, which is an inhibitor of bromodomain-containing protein 4 (Brd4), a partner protein of CDKs, on NB models. In the first paper, the authors demonstrated that this association shows potent activity in human cell lines, and in a xenograft model of NB with telomerase reverse transcriptase (*TERT*) gene overexpression, which is a parameter indicating a poor prognosis [52]. In the second article, the authors noticed that dinaciclib-AZD5153 combination has anticancer activity also in MYCN-amplified and TERT-overexpressing NBs. In fact, this association reduces tumor volume in orthotopic ST16 patient-derived xenografts in mice [53].

As emerged from the CDK2-Dinaciclib complex crystal (PDB code: 4KD1) [54], the pyrazolopyrimidine core strongly interacts with the hinge region of the ATP binding site through a complex hydrogen-bonding pattern. Moreover, a set of hydrophobic interactions contributes to stabilizing the complex, providing inhibitors with high potency and selectivity [55].

#### 2.1.5. THZ1

THZ1 (**6** in Figure 2) is a potent CDK7 inhibitor that possesses an IC_50_ value of 3.2 nM for CDK7 and, also, inhibits CDK12 and CDK13 and shows antiproliferative activity on different cancer cell lines. Remarkably, it was demonstrated to reduce MYCN expression [56]. Tee et al. tested THZ1 in MYCN-amplified NB cells in combination with two potent TK inhibitors, ponatinib, and lapatinib. The compound, when used alone, does not have a significant antiproliferative effect, while acts in synergism with TK inhibitors on MYCN-NB cells, leading to apoptosis and only little effects on healthy cells. The analysis of gene expression demonstrated that the protein kinase phosphatase 1 nuclear targeting subunit (PNUTS) is one of the most down-expressed genes due to the association, leading to a reduced expression of N-Myc protein in cells, even if this effect is not accompanied by a decreased N-Myc mRNA expression [57].

THZ1 contains the typical substituted 2-aminopyrimidine core with a 3-indole in C4. The compound also bears a cysteine-reactive acrylamide moiety [58], fundamental for the high rate of CDK7 selectivity [56,58].

### 2.2. Aurora Kinases (AURKs) Inhibitors 

The family of AURKs includes AURK A, B, and C, STKs that regulate mitosis and are involved in multiple signaling pathways. Amplification of AURKA and AURKB has been related to tumorigenesis in different cancers, including NB [59]. AURKA is associated with MYCN amplification and disease relapse. It has been reported that this kinase regulates the turnover of the N-Myc protein by counteracting its degradation [60]. Furthermore, some studies confirmed that AURKA stabilizes N-Myc protein, while this stabilization could be disrupted by AURKA inhibitors. Richards et al. determined the crystal structure of the complex AURKA-Myc and established that AURKA inhibitors such as alisertib (see next paragraph) made incompatible the binding of Myc to AURKA. This study shed light on the mechanism of action of these compounds in NB cells [61]. AURKA significantly correlates with HR, unfavorable histology, MYCN amplification, and disease relapse in NB patients [62].

#### 2.2.1. MLN8054 and Alisertib

The tricyclic ATP-competitive, reversible AURK inhibitor MLN8054 (**7** in Figure 2) has been developed by Takeda (previously Millennium Pharmaceuticals) for the treatment of different tumors [63]. Among them, potent antiproliferative effects on many NB cell lines in which induces apoptosis [62], were also reported.

Brockmann et al. reported that MLN8054 and its derivative MLN8237, named alisertib, (**8** in Figure 2), disrupt the complex formed between AURKA and N-Myc and promote the degradation of N-Myc. This activity correlates with tumor regression and prolonged survival in a MYCN-driven NB model in mice [64]. Both the compounds bear a benzo[*c*]pyrimido[4,5-*e*]azepine core with a chlorine atom on C9, a substituted phenyl ring in C7, and an aniline in C2.

Alisertib, by Takeda, is an oral AURKA inhibitor that is being investigated for the treatment of different malignancies [65]. It shows activity on some NB cell lines [66] and has usually been used combined with other anticancer agents in different NB models. The results of a phase I and a subsequent phase 2 clinical trials on alisertib in combination with irinotecan and temozolomide for patients with relapsed or refractory NB were reported in 2016 [67] and 2018 [68], respectively. In the phase I trial, the patients received alisertib per os at doses of 45, 60, and 80 mg/m^2^ per day on days 1 to 7 along with irinotecan 50 mg/m^2^ i.v. and temozolomide 100 mg/m^2^ orally on days 1 to 5. The authors performed a dose-escalation study and determined 60 mg/m^2^ per day as the maximum tolerated dose. The toxic effects were thrombocytopenia, neutropenia, diarrhea, and nausea. The progression-free survival rate at 2 years was 52.4% in patients who received the maximum dose. The subsequent phase 2 trial was performed to confirm the phase study 1 results, to evaluate an alisertib oral solution and identify biomarkers of clinical outcomes. The anticancer activity of the previously reported combination was confirmed. The estimated progression-free survival (PFS) at 1 year was 34%. Alisertib oral solution at 45 mg/m^2^ had a significantly better bioavailability compared with the tablets at 60 mg/m^2^. Higher alisertib concentration showed too high hematological toxicity. Finally, it was confirmed that MYCN amplification is related to inferior PFS. 

Alisertib was used in combination with YK-4-279. This compound is an inhibitor of ETV1, a protein belonging to the ETS (erythroblast transformation specific) family, which is implicated in cancer progression. Apart from EVT1 inhibition, the compound seems to act by inducing mitotic arrest in prometaphase, which results in cell death. The association of YK-4-279 and alisertib has a synergistic antiproliferative activity on some NB lines [69].

Currier et al. reported that the association of alisertib with HSV1716, an oncolytic virus that selectively destroys cancer cells, possesses a synergistic antitumor activity in a xenograft NB model derived from SK-N-AS NB cells [70].

Another promising association containing alisertib for NB treatment has been reported by Felgenhauer et al., who demonstrated a synergistic effect of the compound in combination with the BRD4 inhibitor I-BET151 in four xenograft models of HR-NB. The authors reported that this synergy is due, at least in part, to the ability of I-BET151 to reduce a reflexive upregulation of c-Myc in response to AURKA inhibition [71]. 

Very recently, Yang et al. observed that alisertib actually blocks cell division and tumor progression in NB models by inhibiting AURKA activity, but it does not reduce the AURKA level. The authors performed an in vivo study on a xenograft model derived from NB IMR32 cells, by transfecting the cells with AURKA microRNA (miRNA), which reduced AURKA levels. The combination of alisertib with AURKA miRNA results in cell senescence, cell cycle arrest, and destabilization of MYCN, and causes a potent inhibitory effect on NB cell growth in vivo. The work also evidenced that alisertib treatment followed by AURKA miRNA leads senescent cells into apoptosis via inhibition of the Akt/Stat3 pathway [72].

As regards the key motifs for the interaction with AURKA, a cocrystal with MLN8054 (PDB code: 2X81) [73] elucidates the localization of the binding site in the nucleotide pocket of the kinase [74].

#### 2.2.2. Tozasertib

Tozasertib (VX680, MK-0457) (**9** in Figure 2) is a pyrimidine derivative acting as an ATP-competitive pan-AURK inhibitor, with IC_50_ of 0.6, 18, and 4.6 nM for AURK A, B, and C, respectively. It displays activity in the nanomolar range in drug-resistant NB cells [75]. 

An analysis of transcriptome profiles of HR-NB patients, performed by Hsieh et al., confirmed that AURKs are negative prognostic factors and are correlated with MYCN amplification. The authors also reported that tozasertib reduces proliferation, migration, and invasion in MYCN-amplified cells and prolongs the life of mice in xenograft models derived from these cells. Then, the authors identified 150 differentially expressed proteins after tozasertib treatment in the MYCN xenograft mouse model, by using quantitative proteomics. This study showed that tozasertib alters metabolic processes, and in particular indicated a negative correlation of Acyl-CoA dehydrogenase medium chain (ACADM), which is a mitochondrial flavoenzyme involved in fatty acid oxidation, with AURKA and AURKB [76].

As observed in the Tozasertib-AURKA complex (PDB code: 3E5A) [77], the compound is well inserted into the active site, binding a hydrophobic pocket, in the or “closed” AURKA conformation [78].

#### 2.2.3. Barasertib

The hydroxyquinazoline-bearing barasertib (AZD1152-HQPA) (**10** in Figure 2) is a selective AURKB inhibitor [79], which has been tested for different malignancies. Zekri et al. reported that barasertib has antiproliferative and antitumor effects in NB models. In NB cells, the compound upregulates a relevant number of cancer-related miRNA that are tumor suppressors involved in angiogenesis, cell invasion, and metastasis, while it downregulates two miRNA with oncogenic activity [80].

### 2.3. PLK Inhibitors

Polo-like kinase 1 (PLK1) is a STK involved in cell cycle regulation, mitosis, and genomic stability [81]. It is highly expressed in different pediatric solid cancers, including NB, and is correlated to a poor prognosis [82].

PLK1 stabilizes some oncoproteins, including MYC, MYCN, and PAX3-FOXO1, as confirmed by the observation that PLK1 knockdown leads to DNA damage in NB cells with MYCN amplification [83].

#### 2.3.1. Volasertib

**Figure 3:**
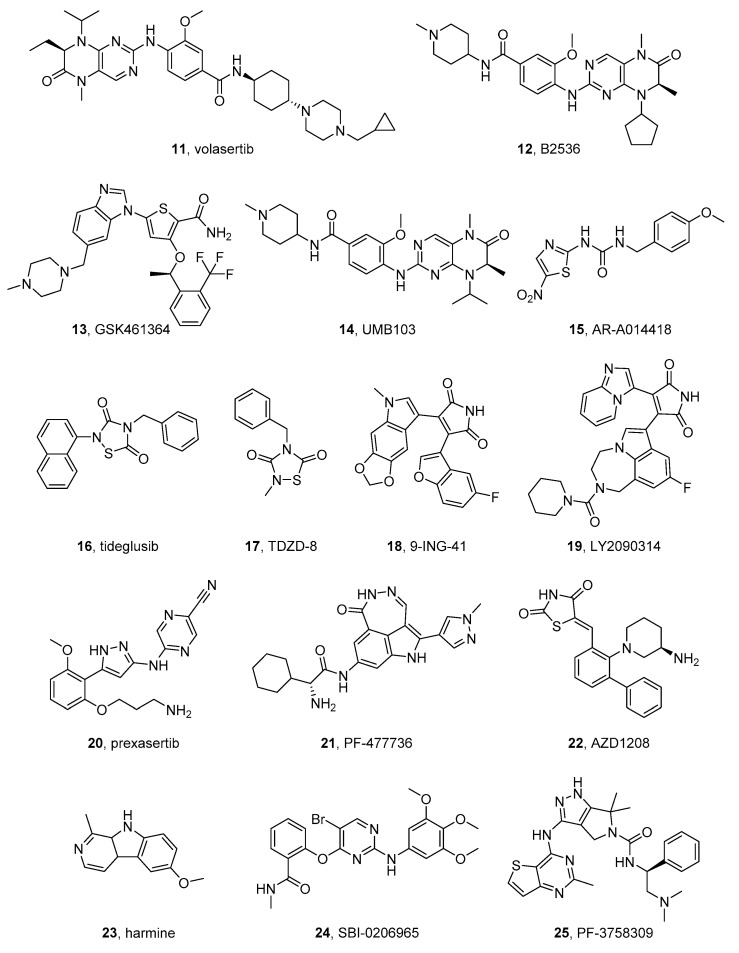
Other STK inhibitors.

Volasertib (BI 6727) (**11** in Figure 3), by Boehringer Ingelheim, is a dihydropteridinone-derived, ATP-competitive kinase inhibitor of PLK1, active in preclinical studies on various cancers and is being tested as anticancer agent in many clinical trials [84]. It has IC_50_ values of 15.5, 34.5, 24.6, and 6.0 nM on NB1643, NB-EBc1, CHLA-90, and CHLA-136 NB cell lines, respectively. In preclinical studies, volasertib shows antiproliferative activity in xenograft models of NB [85].

Analyzing the complex of volasertib bound to PLK1 (PDB code: 3FC2) [86], the dihydropteridinone core results to be involved in two hydrogen bondings in the hinge region. These interactions place the inhibitor in the ATP-binding pocket of the enzyme. Additionally, the basic benzamide-derived moiety, selected to improve the pharmacokinetic properties of the dihydropteridinone, fits in the solvent-exposed region [84].

#### 2.3.2. BI2536

BI2536 (**12** in Figure 3) is a volasertib analogue that is active as PLK1 inhibitor and is in a clinical trial for different malignancies [87].

In 2011, Ackermann et al. investigated its activity in vitro and in vivo against NB. The authors confirmed a high PLK1 expression in HR-NB cells and models. BI 2536 showed antiproliferative activity on all the treated NB cells and antitumor activity in SCID mice bearing IMR-32 and SK-N-AS xenografts [83]. Later, other authors showed that BI2536 decreases NB cell growth and increases apoptosis, and in association with the cytotoxic agent topotecan, further reduces NB survival [88].

Very recently, Li et al. confirmed that PLK1 is expressed in the majority of MYCN amplified (KELLY, SK-N-BE(2), NGP, and KP-N-NS) and non-amplified (SH-SY5Y, SK-N-SH, and NBL-S) NB cells, and that PLK1 expression is not dependent on MYCN amplification. Then, the authors obtain further insights into the mechanism of action of BI2536 and reported that the compound affects cell cycle progression, induces apoptosis by regulating apoptosis-related genes, and reduces autophagy via inhibition of AMPKalfa (AMP-activated protein kinase alfa) in NB cells [89].

A cocrystal of BI2536 and PLK1 (PDB code: 2RKU) [90] shows a reasonably perfect matching between the inhibitor and the binding site thanks to several specific interactions, such as the complex hydrogen-bonding pattern established by the pteridinone carbonyl group and the amide linker of the inhibitor. The latter, along with the piperidine ring, can also interact with the enzyme through Van der Waals contacts, improving its affinity [91].

#### 2.3.3. GSK461364

GSK461364 (**13** in Figure 3) [92] is an ATP-competitive inhibitor of PLK1 endowed with a benzimidazole thiophene scaffold. Its activity is investigated both in in vitro and in vivo models of NB by two different research groups.

As previously reported for other PLK1 targeted compounds, this molecule shows antiproliferative effects and induces apoptosis in NB cells, independently from MYCN expression. Moreover, Pajtel et al. reported that GSK461364 possesses strong antitumor activity in the xenograft model of NB in SCID mice, in which the compound increases the survival time [93].

#### 2.3.4. UMB103

Liu et al. prepared a series of tetrahydropteridine derivatives active as dual inhibitors of PLK1 and BRD4, which could be useful in the treatment of cancer [94]. Later, Timme et al. tested a combination of PLK1 inhibitors (Volasertib or GSK461364) with a BRD4 inhibitor (MK-8628). The combinations showed a synergistic behavior on NB cells, independently of MYCN expression levels. Furthermore, the authors tested some of the dual inhibitors synthesized by Liu, and identified compound UMB103 (**14** in Figure 3) as a potent antiproliferative agent against NB, both in vitro and in vivo, where it leads to a significant tumor size reduction in patient-derived xenograft models [95].

### 2.4. GSK-3 Inhibitors

Glycogen synthase kinase 3 (GSK-3) is a STK. When it was discovered, it was indicated as a regulator of glycogen synthase. Successively, it has been demonstrated to be involved in the regulation of many pathways. In mammals, it is present in two isoforms (GSK-3α and GSK-3β) and has relevant roles in many diseases, including Alzheimer’s disease, type 2 diabetes, inflammation, and cancer [96]. Moreover, some studies indicate the involvement of -GSK-3 in NB [97] and consequently, its inhibitors have been tested in preclinical models of this disease.

#### 2.4.1. AR-A014418

Carter et al. reported the antiproliferative effects of AR-A014418 (**15** in Figure 3), an ATP-competitive GSK-3 inhibitor, on NGP and SH-5Y-SY NB cell lines. Structurally, AR-A014418 is a small molecule with a central *N*,*N*’-disubstituted urea bearing a thiazole and a benzyl substituent. The compound reduces more potently GSK-3α phosphorylation at Tyr279 compared to GSK-3β phosphorylation at Tyr216. The authors also observed that this specific GSK-3α inhibition leads to the reduction in neuroendocrine markers, indicating a possible anti-cancer role for GSK-3α inhibitors [98].

A cocrystal structure of AR-A014418 complexed to GSK-3β (PDB code: 1Q5K) [99] is available and shows the binding of the compound to the hinge/linker region through three hydrogen bond interactions. Moreover, the nitro group fits the inner part of the ATP pocket, while its phenyl ring is involved in a π-guanidinium stacking with an arginine residue belonging to the kinase [100].

#### 2.4.2. Tideglusib

Tideglusib (**16** in Figure 3) is a derivative in which the urea function of AR-A014418 has been included in a 1,2,4-thiadiazolidine-3,5-dione ring. It is a non-reversible GSK-3 inhibitor recently designed as an “Orphan Drug” to target Alzheimer’s disease and is being tested in clinical trials for this pathology. Duffy et al. reported that the compound reduced the viability of human IMR32 NB cells [101].

Mathuram et al. studied the effects of tideglusib on the same cell line, using lithium, a known GSK-3 inhibitor, as the reference compound. Tideglusib causes a significant dose-dependent increase in apoptosis, decreases colony formation, and increases the level of sub-G0/G1 population in IMR32 cells. This investigation indicates tideglusib as a promising apoptotic inducer in human NB IMR32 cells [102]. Very recently, Bahmad et al. demonstrated that the compound has antiproliferative activity on three human NB cell lines, SK-N-SH, SH-SY5Y, and IMR-32. Importantly, tideglusib reduces the self-renewal ability of slowly replicating cancer stem cells (CSCs), which are resistant to many therapeutic agents. Moreover, the compound shows anticancer activity in vivo in NB models. Its activity is due to the inhibition of GSK-3β, which is able to sustain the survival of CSCs and make them insensitive to cytotoxic agents and radiation therapy [103].

#### 2.4.3. 9-ING-41

Ugolkov et al. found GSK-3β expression in 34 out of 51 examined cases (67%) of NB. Three GSK-3 inhibitors, AR-A014418, TDZD-8 (**17** in Figure 3), and 9-ING-41 (**18** in Figure 3), suppressed the growth of NB cells. In particular, the maleimido ATP-competitive GSK-3β inhibitor, 9-ING-41, possesses a wide antitumor activity in preclinical models. It results to be the most active compound and has a synergistic effect with CPT-11 (irinotecan, a well-known camptothecin derivative acting as topoisomerase inhibitor) in reducing tumor volume in mouse xenograft models. The inhibition of GSK-3 by these inhibitors resulted in decreased expression of the antiapoptotic molecule XIAP and increased apoptosis in NB cells [104].

#### 2.4.4. LY2090314

LY2090314 (**19** in Figure 3) is bis(aryl)maleimide compound endowed with a potent inhibitory activity on GSK-3 with IC_50_ values of 1.5 nM and 0.9 nM for GSK-3α and GSK-3β, respectively. It is in clinical trials for the treatment of malignancies [105].

Kunnimalaiyaan et al. investigated the effect of LY2090314 on the three NB cell lines NGP, SK-N-AS, and SH-SY-5Y. These lines have different characteristics: SH-SY-5Y cells bear non-amplified MYCN and the ALK mutation F1174 L, NGP cells bear MYCN amplified and ALK wild type, and SK-N-AS bear non-amplified MYCN and wild-type ALK. The authors compared the compound’s activity with that of the previously reported GSK-3 inhibitor tideglusib. LY2090314 demonstrated antiproliferative activity on the three cell lines, starting from a 20 nM concentration, induced apoptosis, and, also, the level of cyclin D1, a protein that plays fundamental roles in cell cycle and apoptosis regulation. This study led to the observation that LY2090314 effectively reduces the growth of both human MYCN amplified and non-amplified NB cell lines in vitro and further suggests GSK-3 as a therapeutic target for NB [106].

### 2.5. Checkpoint Kinase 1 and 2 (CHEK1-2) Inhibitors

CHEK1-2 are STK involved in DNA repair and cell cycle regulation. It has been widely demonstrated that inhibitors of these kinases have anticancer action. Moreover, they increase the DNA-damaging effects of cytotoxic therapies [107].

#### 2.5.1. Prexasertib

Prexasertib (LY2606368) (**20** in Figure 3) is a substituted pyrazinecarbonitrile CHEK1 inhibitor, with a lower activity against CHEK2. The compound was developed by Eli-Lilly for the treatment of some tumors, where it induces DNA double-strand breaks causing apoptosis. Lowery et al. evaluated the activity of this compound in preclinical models of NB. The compound shows antiproliferative activity on NB cells and in different xenograft mouse models. It promotes double-strand breaks leading to the death of NB cells. By silencing CHEK1 and/or CHEK2, the authors demonstrated that the compound activity is due to selective CHEK1 inhibition [108]. The same authors reported that the combination of prexasertib with classic cytotoxic chemotherapy has a synergic antitumor activity in preclinical models of NB and other pediatric cancers [109].

#### 2.5.2. PF-477736

PF-477736 (**21** in Figure 3) by Pfizer is a diazapinoindolone compound, active as an ATP-competitive inhibitor of CHEK1 and shows a *K_i_* of 0.49 nM in enzymatic assays, with a selectivity of 100-fold for CHEK1compared to CHEK2. It inhibits also VEGFR2, AURKA, FGFR3, Flt3, Fms, Ret, and Yes. PF-477736 is active in different models of solid tumors and is currently being evaluated in clinical trials [110].

Ando et al. investigated the activity and the mechanism of action of PF-477736 on MYCN-amplified NB cell lines. The compound increases the expression of two pro-apoptotic proteins, BAX and PUMA in two sensitive NB cell lines, SMS-SAN and CHP134, providing a mechanism for the effect of CHEK1 inhibition. On the other hand, it is poorly active in NB-39-nu and SK-N-BE cell lines, where it activates the ataxia-telangiectasia mutated STK (ATM)-p53-p21 axis of the DDR pathway, which renders the cells quite resistant to PF-477736. Importantly, the association of PF-477736 and the ATM inhibitor Ku55933 has antiproliferative effects also on NB-39-nu and SK-N-BE cells [111]. 

### 2.6. PIM Inhibitors

Human PIM (proviral integration site for Moloney murine leukemia virus) kinases constitute a family of STKs that possesses several biological functions in cell survival, proliferation, and differentiation, and its overexpression has been observed in several human cancers. Indeed, PIM kinases are proto-oncogenes that have been implicated in early transformation and tumor progression. For these reasons, Pim kinases are emerging as important targets in drug discovery, and many Pim inhibitors have been reported in the last few years [112]. 

#### AZD1208

PIM inhibition has been suggested as a therapeutic option in MYC/MYCN-associated tumors. Indeed, it has been reported that high PIM kinase expression is correlated with poor overall survival in these malignancies. AZD1208 (**22** in Figure 3) is a potent ATP-competitive pan-PIM inhibitor endowed with a benzylidene-1,3-thiazolidine-2,4-dione core that was studied in the treatment of NB cell lines, where it was found to suppress the proliferation, by inhibiting mTOR signaling. Moreover, the compound impaired the growth of NF1 wild-type xenografts [113].

Trigg et al. identified a high expression of the PIM gene in ALK-positive NB cells and reported that knockdown of PIM sensitizes NB cells to ALK inhibitors, independently from MYCN expression. In xenograft models derived from cells of an HR-NB patient with ALK mutations, the association of the PIM inhibitor AZD1208 and ceritinib shows significantly enhanced anti-tumor activity compared with the two agents used alone [114].

### 2.7. DYRK Inhibitors

Dual-specificity tyrosine phosphorylation-regulated kinases (DYRKs) are a conserved family of eukaryotic STKs, related to CDKs, mitogen-activated protein kinases (MAPKs), GSKs, and CDK-like kinases (CLKs), which are collectively indicated as the CMGC group.

DYRKs are a family including four members (DYRK1A, DYRK1B, DYRK2, and DYRK4), which are involved in cancer, as they can act as regulators of protein stability during the progression of the cell cycle and regulate the activity of the proteasome [115]. 

#### Harmine

Harmine (**23** in Figure 3) is a tricyclic β-carboline alkaloid isolated from the plant Peganum harmala and is known to inhibit all DYRK family members in an ATP competitive fashion [116]. It induces apoptosis and inhibits cell proliferation, migration, and invasion in a dose-dependent manner in many human cancer cell lines [8], Uhl et al. tested the antiproliferative activity of this natural compound on four human NB cell lines, in particular SKNBE and KELLY (MYCN-amplified), and SKNAS and SKNFI (MYCN-non-amplified). The IC_50_ values for harmine after 72 h from the treatment were 169.6, 170.8, and 791.7 μM for SKNBE, KELLY, and SKNFI, respectively, and a pro-apoptotic activity is determined. The authors generated a computational model of harmine bound to DYRK2 by using known X-ray structures and demonstrated that harmine can bind to DYRK2. Moreover, they found an increased DYRK2 mRNA expression in a consistent number of NB patients. All these results indicate the use of harmine as a potential drug for MYCN-amplified NB [117].

DYRK1A–harmine cocrystal (PDB code: 3ANR) [118] shows the presence of the inhibitor in the ATP-binding pocket. Its orientation allows the establishment of crucial hydrogen bonds of the pyridine nitrogen and the methoxy oxygen with highly conserved residues in the hinge region [116].

### 2.8. Unc-51 Like Autophagy Kinase 1 (ULK1) Inhibitors

The STK ULK1 is involved in autophagy, a degradation process that maintains cellular homeostasis through the elimination of dysfunctional proteins and organelles [119]. Importantly, many tumors become dependent on autophagy since it generates metabolic fuel and reduces oxidative stress [120]. ULK1 constitutes an optimal target for autophagy inhibition, and ULK1 inhibitors are being tested as anticancer agents, showing interesting results on some types of cancers [121].

#### SBI-0206965

The ULK1 inhibitor SBI-0206965 (**24** in Figure 3) with a pyrimidine scaffold has an IC_50_ of 108 nM on the kinase [122]. Although the role of autophagy in NB growth and metastasis is still unclear, it has been reported to exert antiproliferative and proapoptotic effects on SK-N-AS, SH-SY5Y, and SK-N-DZ NB cell lines [123]. 

### 2.9. PAK4 Inhibitors

P21-activated kinase 4 (PAK4) is a STK that is involved in different biological activities, including embryonic and neural development, and premature cell senescence. More recently, this enzyme has also been shown to be involved in many human cancers [124].

#### PF-3758309

Murray et al. reported that PAK4 is overexpressed in NB and correlates with a poor prognosis, as demonstrated by the analysis of PAK4 expression in NB specimens which were obtained from 50 NB patients. Intending to demonstrate the involvement of PAK4 in NB, the authors used the pan-PAK inhibitor PF-3758309 (**25** in Figure 3) [125], which shows apoptotic activity and leads to cell cycle arrest at the G1 phase in SH-SY5Y and IMR-32 NB cells. This work is the first demonstration of PAK4 involvement in NB and opens the way to further investigation on PAK inhibitors for a potential NB treatment [126].

The complex of PF-3758309 with PAK4 (PDB code: 2X4Z) [127] shows the compound fitting in the ATP-binding site: multiple hydrogen bond interactions are observed between the pyrrolopyrazole core and the amine linker of the thienopyrimidine ring with the hinge region. A key water bridge is generated by the urea carbonyl oxygen, a conserved water molecule, and charged residues. Furthermore, the dimethylamine moiety establishes a strong charge-charge interaction with an aspartate residue (relevant contact for potency). Multiple hydrophobic interactions are generated by the thienopyrimidine cycle (essential for potency), the dimethyl group of the pyrrolopyrazole, and the phenyl ring (fundamental for selectivity) [125].

### 2.10. PI3K/AKT/mTOR Inhibitors

PI3K/AKT/mTOR signaling pathway includes two STKs (mammalian target of rapamycin, Mtor, and AKT), and an additional kinase that phosphorylates the 3-hydroxyl group within the inositol ring of phosphoinositides, phosphoinositide 3-kinase (PI3K). mTOR is involved in many cell functions, including growth, proliferation, apoptosis, and autophagy and its hyperactivation has been detected in several human cancers, thus representing, together with its upstream effectors, an important target for cancer therapy [128].

Studies on NB preclinical models have confirmed a role for the PI-3K/AKT/mTOR pathway in NB pathogenesis [129]. Furthermore, inhibitors of this pathway have anti-tumor effects and lead to improved survival rates in a preclinical model of NB.

#### 2.10.1. Dactolisib

**Figure 4:**
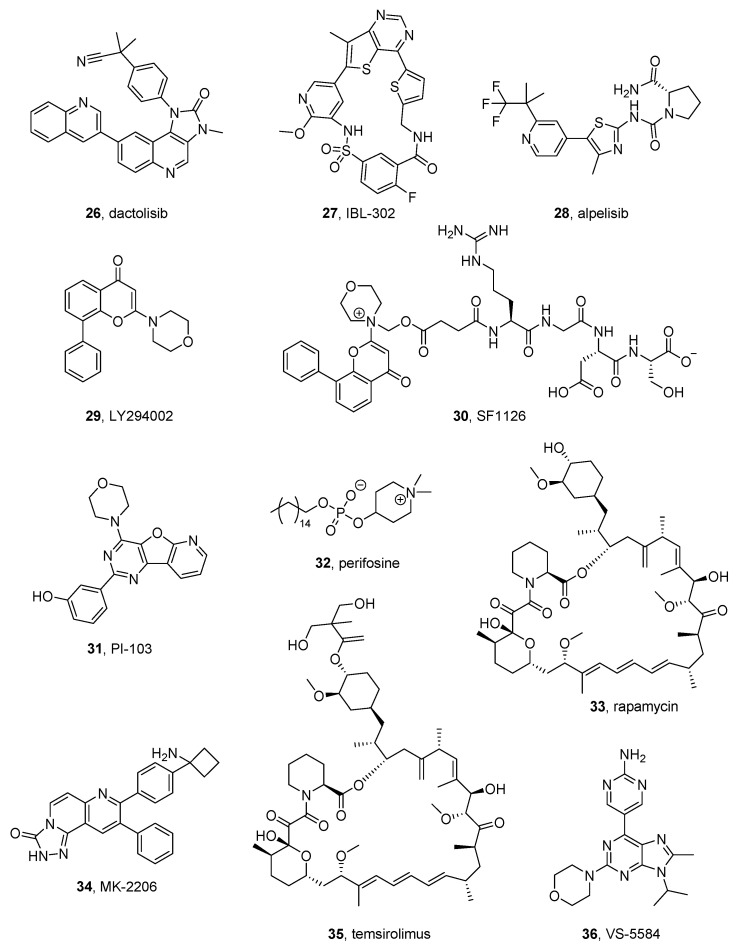
PI3K/AKT/mTOR inhibitors.

Dactolisib (NVP-BEZ235, BEZ-235, RTB101) (**26** in Figure 4) is an imidazoquinazoline that inhibits both PI3K and mTOR and is being tested in clinical trials for the treatment of different tumors. This compound was tested in combination with chloroquine on NB cells and this association synergically induces apoptosis, [130] and in combination with the anti-GD2 ganglioside (GD2) 14G2a mouse monoclonal antibody, enhancing the cytotoxic effects against IMR-32, CHP-134, and LA-N-1 NB cells [131]. Moreover, dactolisib was tested in association with oridoin, a natural compound extracted from Rabdosia rubescens, resulting in the induction of apoptosis and the enhancement of autophagy in NB cells. Moreover, it reduces NB xenograft growth in a more effective way than either agent alone [132].

Vaughan et al. demonstrated that dactolisib kills MYCN-expressing NB cells, and induces apoptosis of transgenic MYCN-driven NB, by degrading the MYCN protein. The authors explain its activity as a result of the inhibition of mTOR but not of that of PI3K [133].

Liu et al. reported that the dactolisib blocks SH-SY5Y and SK-N-MC NB cell growth with EC_50_ values in the nanomolar range and observed the reduction of the cyclins D1 and E1 due to the activation of GSK3beta [134].

Recently, Kostopoulou et al. demonstrated that PI3Kα and FGFR3 mutations are present only in a few NB samples that were found susceptible to dactolisib, especially when used in association with a FGFR3 inhibitor (see FGFR inhibitor paragraph) [135].

Unfortunately, either intrinsic or acquired resistance to PI3K inhibitors represents a potential obstacle to effective therapy. Resistance mechanisms include activation of downstream mTOR complexes and activation of other signaling pathways. For example, an increased PIM expression has been linked to PI3K inhibitor resistance.

#### 2.10.2. IBL-302

Mohlin et al. synthesized a series of PIM/PI3K/mTOR triple kinase inhibitors. The most active compound of the series, IBL-302 (**27** in Figure 4), inhibits PI3Kα, mTOR, PIM1, PIM2, and PIM3 with IC_50_ values of 2.08, 81.3, 22.8, 7.74, and 5.86 nM, respectively. The authors tested the compound on many cancer cell lines and found that it has antiproliferative activity on PDX and SK-N-BE(2)c NB cells. Moreover, IBL-302 induces apoptosis in NB patient-derived xenograft models and increases the activity of classic chemotherapeutic agents, such as cisplatin, doxorubicin, and etoposide. These results indicate the possible use of IBL-302 in combination with cytotoxic drugs for NB therapy [136].

Structurally, IBL-302 is a macrocycle containing several heterocycles, such as thieno[3,2-*d*]pyrimidine, pyridine, thiophene, and phenyl rings.

#### 2.10.3. Alpelisib

The substituted aminothiazole alpelisib (NVP-BYL719) (**28** in Figure 4), by Novartis, is a potent and selective PI3Kα (a member of the PI3K family) inhibitor with IC_50_ of 5 nM in enzymatic assays and it maintains activity on the oncogenic mutated PI3Kα, that, although found in many tumors, are not very frequent in NB [135]. 

The association of alpelisib with fulvestrant was approved by FDA and EMA in 2019 and 2020, respectively, for the treatment for hormone receptor (HR)-positive, human epidermal growth factor receptor-2 (HER2)-negative breast cancer in patients with a PIK3CA (the p110α subunit of PI3K) mutation [137]. 

Holzhauser et al. tested the efficacy of alpelisib and erdafitinib, a FGFR inhibitor, on 5 different NB cell lines. The compounds were tested alone and in combination with cisplatin, vincristine, or doxorubicin. Monotherapy with alpelisib or erdafitinib inhibits NB cell proliferation in a dose-dependent manner, while their association is more active. However, the combination of alpelisib or erdafitinib with usual cytotoxic agents leads to variable effects, from synergistic to antagonistic [138].

#### 2.10.4. LY294002 e SF1126

LY294002 (**29** in Figure 4), is a benzopyran-4-one that acts as a multitargeted kinase inhibitor, strongly inhibiting PI3K. In the past, it was tested in many cancers, including NB.

SF1126 **(30** in Figure 4) is a prodrug of LY294002 conjugated to RGDS tetrapeptide with the morpholino nitrogen. This amino acid sequence is useful to target the active moiety (LY294002) to integrin-expressing tissues. This prodrug resulted to be effective in preclinical models of NB [139]. In this compound, the active moiety of LY294002 is linked to an RGDS tetrapeptide that directs the active agent to integrin-expressing tissues. More recently, Erdreich-Epstein et al. reported that the compound also inhibits BRD4 and possesses antiproliferative activity in the MYCN amplified NB cell line IMR-32. Moreover, SF1126 reduces MYCN expression in IMR-32 and CHLA136 cells, leading to an overall decrease in cell viability. Finally, the compound inhibits tumor growth in a xenograft model of NB [140]. 

By observing the LY294002-PI3K complex (PDB code: 1E7V) [141], the morpholino oxygen of the inhibitor interacts with the backbone PI3K. However, it does not extend into the space usually occupied by the ATP phosphate groups but into the hydrophobic region I, located in the opposite place with respect to the ribose binding region [142].

#### 2.10.5. PI-103

The pyridofuro[3,2-*d*]pyrimidine PI-103 (**31** in Figure 4) is a potent PI3K and mTOR inhibitor with IC_50_ values of 8, 88, 48, 150, 20, and 83 nM for p110α, p110β, p110δ, and p110γ PI3K subunits, mTORC1, and mTORC2, respectively. PI-103 also inhibits DNA-dependent protein kinase (DNA-PK) with an IC_50_ of 2 nM. PI-103 shows synergic activity with different cytotoxic agents, including doxorubicin, etoposide, topotecan, cisplatin, vincristine, and taxol in NB cells, where the combinations induce apoptosis. In particular, the association of PI103 and doxorubicin suppresses tumor growth in NB cells derived from patients and is active in an in vivo NB model [143]. More recently the activity of the association of PI-103 and doxorubicin was confirmed on SH-SY5Y and SK-N-BE(2) both in vitro and in vivo. In NB xenograft models it leads to improvement in mice survival [144]. 

#### 2.10.6. Perifosine

Perifosine (**32** in Figure 4) is an hexadecanyl-phospholipid with a dimethyl piperidinium group developed by Keryx Biopharmaceuticals. It is an allosteric pan-AKT inhibitor and, also, inhibits PI3K. Currently, the compound is being tested in clinical trials for the treatment of different solid tumors. It reached the status of orphan drug in the USA for the treatment of multiple myeloma and NB and multiple myeloma in the EU. In preliminary studies, perifosine demonstrates antiproliferative effects on NB cells both in vitro and in vivo [145] and reduces chemoresistance in NB cells in vivo [146]. 

Perifosine has been tested in several phase I clinical trials, whose results have been published. In detail, it was tested at doses ranging from 25 to 125 mg/m^2^/day for 28 days per cycle on different pediatric tumors. Five NB patients out of 8 achieved a prolonged stable disease (≥11 months) in three cases [147]. In another trial performed on 19 NB Japanese patients with refractory or recurrent disease the response rates and disease control rates were 9% and 55%, respectively. Perifosine monotherapy was well tolerated, but the results were not particularly satisfying [148]. Another study supported the use of perifosine for MYCN-non amplified HR NB as monotherapy, after the treatment with standard treatments [149].

Moreover, perifosine was tested in a phase I clinical trial in combination with the mTOR inhibitor temsirolimus for the treatment of recurrent pediatric tumors. This combination was safe, but unfortunately, no partial or complete responses were achieved [150]. Other preclinical studies obtain further insights into the mechanism of action of perifosine in SK-N-AS cells. Gu et al. reported that in addition to AKT inhibition, the compound alters the acetylation levels of functional proteins [151]. 

Very recently, Le Grand et al. reported that the inhibition of total AKT activity, rather than that of specific isoforms, is fundamental to reducing MYCN expression and decreasing the proliferation of MYCN-amplified NB cells. Moreover, the authors showed that the pan-AKT inhibitor perifosine has a synergic activity with the classic cytotoxic agents, including vincristine, etoposide, and doxorubicin, which are used in NB treatment. These associations are active in MYCN-amplified NB cells. In particular, the combination of perifosine and vincristine has synergistic activity in vivo, with a significantly prolonged median survival in MYCN-amplified BE(2)-C tumor-grafted mice [152]. 

#### 2.10.7. Rapamycin and MK-066

Regarding mTOR inhibitors, early studies determined that rapamycin and MK-066 (**33** and **34**, respectively, in Figure 4), were active in NB models [153].

The macrolide rapamycin (sirolimus) was isolated from the bacterium *Streptomyces hygroscopicus* and exerts immunosuppressant and antiproliferative activities, inhibiting mTOR. It was approved as an anti-rejection drug in 1999 and has been tested alone and in combination with other anticancer agents in many malignancies. Phases I and II clinical trials indicated some clinical benefit in NB patients treated with the combination of rapamycin and vinblastine, resulting in a prolonged stable disease in patients with relapsed NB [154].

More recently Lin et al. reported that rapamycin reduces proliferation and stimulates autophagy in NB cells. The compound blocks the cell cycle at the G_0_/G_1_ phase and reduces the expression levels of P62, mTOR, and phospho-mTOR proteins [155].

Bahmad et al. investigated the involvement of the AKT/mTOR pathway in the activity of CSCs, which are involved in the recurrence of NB and other cancers. The authors used rapamycin to assess the role of the inhibition of the AKT/mTOR pathway in vitro on NB SH-SY5Y cells, and demonstrated that it slightly decreases the survival of SH-SY5Y cells in a 2D model, while the antiproliferative effect is more pronounced in a 3D culture model. Moreover, rapamycin decreases the migratory abilities of the cells and decreased their sphere-forming units by extinguishing their CSC populations [156].

Rapamycin has been very recently associated with MK-066 for preclinical studies on NB. MK-066 is an allosteric inhibitor of AKT and showed preclinical activity in different tumor models [157]. MK-066 has antiproliferative activity at concentrations in the range 5–10 micromolar on sensitive NB cells (LAN-1, KP-NSIFA, NB-19, and SK-N-DZ), while having low activity on resistant cells (LAN-1-MK, KP-N-SIFA-MK, and SK-N-DZ-MK) [158].

Dong et al. tested rapamycin-MK-066 combination on different NB cells and demonstrated that the latter increases autophagy and necroptosis, contributing to NB cell death. The authors also investigated the variation of MYCN level in the treated cells and found that MYCN expression is downregulated in MYCN-amplified cell lines (NGP, BE2) and is upregulated in MYCN non-amplified cell lines (AS, SY5Y). This study obtains further insights into the role of MYCN in MYCN-amplified NB cells [159].

The structures of these compounds have relevant differences, being rapamycin a macrolide, and MK-066 a tricycle endowed with fused triazolo and naphthyridin-3-one rings.

#### 2.10.8. Temsirolimus 

Temsirolimus (CCI-779) (**35** in Figure 4), is a macrolide prodrug of rapamycin approved for the treatment of renal cell carcinoma. Since the treatment with mTOR inhibitors alone did not lead to satisfactory results in NB treatment, temsirolimus has been tested in some preclinical and clinical studies in combination with other agents. 

Moreno-Smith et al. tested temsirolimus in combination with RG7388, an inhibitor of MDM2 (mouse double minute 2 homolog), an enzyme that negatively regulates the p53 tumor suppressor protein and whose inhibition leads to p53 activation. The authors observed a strong antitumor activity using this combination in an orthotopic xenograft model of NB together with a block of tumor regrowth after treatment completion [160]. 

In a phase II clinical trial, temsirolimus was added to the association irinotecan-temozolomide in patients with relapsed or refractory NB. The selected patients were stratified by different disease parameters, including MYCN amplification status. Unfortunately, the association did not lead to positive results to merit further studies [161]. Furthermore, temsirolimus was recently tested in association with pyrrolomycin MP1, a novel marino-pyrrole derivative, which demonstrates activity in a resistant NB cell line overexpressing MYCN. In this cell line, the compound inhibits both MYCN and MCL-1, and stimulates autophagy and inhibition of oxidative phosphorylation [162]. The combination with temozolomide increases the antiproliferative activity of MP1 in MYCN amplified (BE-2c, IMR) NB cells, while having low activity in non-amplified (SKN-AS) NB cells. Moreover, in in vivo studies, the association leads to a complete response in three out of five tumor-bearing mice and slowed tumor growth [162]. 

#### 2.10.9. VS-5584 

VS-5584 (**36** in Figure 4) is a substituted purine derivative recently synthesized, active as a PI3K/mTOR dual inhibitor. It possesses antiproliferative activity on NB cells, where it induces G0/G1 cell cycle arrest. The compound has also antitumor activity in a xenograft mice model [163].

## 3. MEK Inhibitors

MEK is a class of enzymes that includes both STKs and TKs [164]. The enzymes of the RAS-RAF-MEK-ERK pathway, also indicated as MAPK-ERK pathway is hyperactivated in many tumors and frequently presents mutations in refractory NB [165]. 

### 3.1. Trametinib and CH5126766

**Figure 5:**
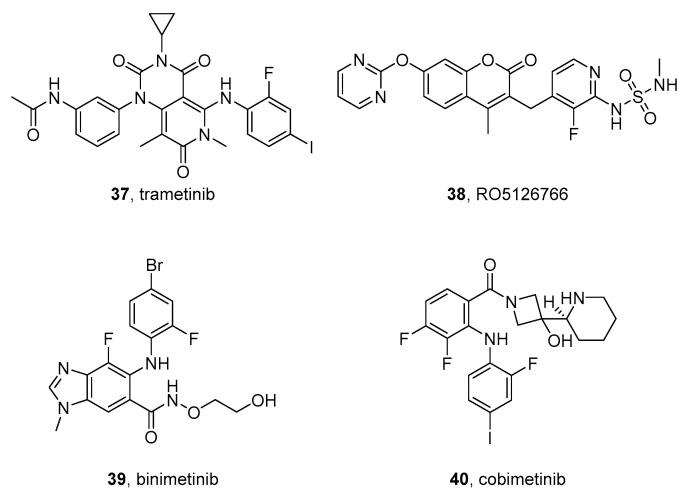
MEK inhibitors.

Trametinib (**37** in Figure 5) is a MEK inhibitor approved for unresectable or metastatic BRAF mutation-positive malignant melanoma and in clinical trials for different cancers.

Tanaka et al. tested trametinib or the RAF/MEK inhibitor CH5126766 (RO5126766) (**38** in Figure 5) on five NB cell lines (HP134, IMR5, NB69, NLF, SK-N-A, used as wild-type or after genetic profile mutation). The authors observed an antiproliferative effect in ERK-active cells and both the compounds inhibited ERK phosphorylation and MYCN expression. RAS/RAF mutations are not always detected in drugs [166]. Successively, Umapathy et al. reported that trametinib inhibits the growth of RAS-mutant NB but not ALK-dependent NB in xenograft models. For these reasons, the authors advise against the apparently rational option of using MEK inhibitors to treat ALK-addicted NB [167]. 

Takeuchi et al. reported that the treatment with trametinib leads to a short-term growth inhibition in CHP-212 and SK-N-AS xenografts, which were ERK-positive before treatment, but in long-term treatment, the xenograft started to regrow, due to the persistence of ERK-positive cells [168]. Very recently, Puissant et al. tested trametinib combination with the bromodomain and extra-terminal (BET) inhibitors I-BET726 or I-BET762 since BET inhibitors have been shown to reduce MYCN expression [169]. The association of MEK and BET inhibitors has synergistic effects in the majority of NB cells, but, unfortunately, has little activity in vivo models, being active only in a non-MYCN-amplified, NRAS-mutated NB xenograft model, while no activity was found in MYCN-amplified models [170]. 

Both trametinib and CH5126766 are characterized by conjugated bicyclic scaffolds, a pyrido[4,3-*d*]pyrimidine-2,4,7-trione and a 2*H*-chromen-2-one, respectively.

### 3.2. Binimetinib 

Benzo[*d*]imidazole-based binimetinib (Mektovi, ARRY-162) (**39** in Figure 5) is a MEK1/2 inhibitor approved in 2018 for the treatment of unresectable or metastatic melanoma with BRAF mutations, in association with encorafenib, a MAPK inhibitor. Woodfield et al. tested binimetinib on primary NB tumor samples and many NB cell lines. Using MTT assays, the authors determined IC_50_ values in the range from 8 nM to 1.16 μM for cells sensitive to binimetinib, while no activity was observed on resistant cells. Interestingly, the cell lines susceptible to binimetinib showed higher endogenous levels of phosphorylated MEK and ERK, determined by western blot assays. Moreover, the authors found that the expression of neurofibromin 1 (NF1) protein is correlated to responses to binimetinib, and suggested that the use of NF1 as a biomarker could be useful to identify patients that may respond to MEK inhibitors [171].

### 3.3. Cobimetinib

The azetidine-containing cobimetinib (GDC-0973, RG7420) (**40** in Figure 5) is a MEK inhibitor by Exelixis and Genentech (Roche) approved by the FDA and EMA in 2015 for the treatment of metastatic melanoma expressing BRAF V600E or V600K mutation as a combination with vemurafenib [172]. The compound has also been tested by Singh et al. on a wide number of NB cell lines, showing antiproliferative activity due to MEK inhibition [173]. 

Cobimetinib in complex with MEK1 (PDB code: 4LMN) [174] shows key interactions of the inhibitor with the kinase backbone along with weaker interactions made by the aromatic fluorine atom [175].

## 4. TK Inhibitors

### 4.1. ALK Inhibitors

ALK is a transmembrane receptor tyrosine kinase (RTK) belonging to the insulin receptor superfamily [176], whose deregulation is involved in the carcinogenesis process of various human malignancies, including NB [177]. ALK cooperates with MYCN in inducing NB tumors in experimental models [178]. This kinase is frequently activated by amino acid mutations, amplification, or, rarely, translocation events. The most common mutations (85%) occur at three positions in the ALK catalytic domain: F1174 (mutated to L, S, I, C, or V), F1245 (mutated to L, I, V, or C), and R1275 (mutated to Q or L) [179]. Other point mutations have been also found and are reported in an updated review [180]. Recently, Javanmardi et al. performed an ultra-deep sequencing in order to determine the frequency of ALK mutations that might be missed with conventional sequencing methods, due to different degrees of sensitivity. The authors found 6 mutations in 16 samples, which could be undetected with previously reported analysis [181]. The identification of ALK mutations, already present at the beginning of the disease or later acquired in NB patients is fundamental in the choice of treatment options and in particular in the use of the right kinase inhibitor.

Many available ALK inhibitors are tested alone or in combination with other anticancer agents for the treatment of NB. They are ATP-competitive type I inhibitors that bind the enzyme active conformation. However, the main issues are represented by the lack of sensitivity of mutated ALKs to monotherapies with ALK inhibitors and the rise of secondary mutations after the treatment. Therefore, combination strategies targeting different enzymes or signaling pathways have been seen as useful approaches to increase the efficacy of ALK inhibitors.

#### 4.1.1. Crizotinib

**Figure 6:**
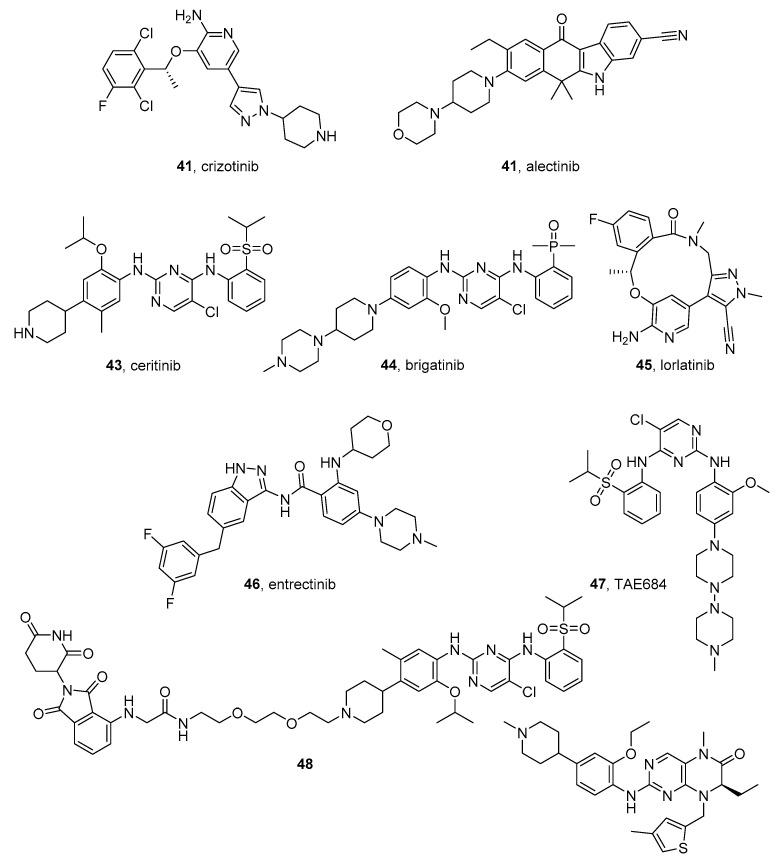
ALK inhibitors.

The type I kinase inhibitor crizotinib (PF-02341066) (**41** in Figure 6) is the first ALK-targeted TK inhibitor (IC_50_ = 24 nM) that entered the clinics [182]. The compound is a dual inhibitor of ALK and ROS1, a RTK structurally similar to ALK. Crizotinib was approved by FDA in 2011 and then by EMA for the treatment of ALK-positive, metastatic non-small cell lung cancer (NSCLC). Currently, it is being tested for treatment of anaplastic large cell lymphoma, NB, and other malignancies [183]. 

Activating mutations and amplifications of ALK leads to crizotinib resistance. NB cell lines with the R1275Q mutation are initially sensitive to ALK inhibition, while cells harboring mutations in the residues F1174 and F1245 are relatively resistant to crizotinib [184]. Bresler et al. reported that the F1174L mutation increases the ATP affinity of ALK and reduces sensitivity to crizotinib [184]. Further clinal studies confirmed that R1275Q-mutated ALK and ALK-amplified NB cells are sensitive to crizotinib, while cells harboring the mutation F1174L ALK are resistant. 

Krytska et al. evaluated the activity of the combination of crizotinib with topotecan or cyclophosphamide in murine models of NB harboring ALK mutations, included those associated with crizotinib resistance. The authors found that the association has a synergistic activity and leads to enhanced tumor regression and event-free survival in mouse models with ALK mutations, compared with the drugs used alone [185]. 

Zhang et al. studied the therapeutic effects of crizotinib and its association with a MEK inhibitor (trametinib) or with low-dose metronomic (LDM) (low dosage of a drug administered daily) topotecan in preclinical NB models bearing the ALKF1174L mutation. The authors tested crizotinib on a wide number of NB cells and reported an activity ranging from 0.25 to 5.58 μM. In detail, ALK-mutated cell lines SH-SY5Y, KELLY, LAN-5, and CHLA-20 were more sensitive than ALK wild-type cell lines. Moreover, the association of crizotinib with and LDM topotecan has a synergistic activity on ALK^F1174L^-mutated SH-SY5Y cells. Crizotinib showed low activity in the xenograft model derived from ALK^F1174L^-mutated SH-SY5Y and KELLY cells, while the combination with topotecan leads to a significant tumor growth delay [186]. 

A computational study highlights the Crizotinib fitting into the ALK ATP-binding pocket thanks to the interaction of the 2-amino pyridine ring within the adenine site. Furthermore, the piperidine ring protrudes toward the solvent-exposed surface and a hydrophobic interactions network stabilizes the inhibitor in the *N*-terminal lobe [187]. 

#### 4.1.2. Alectinib

Alectinib (**42** in Figure 6) is a potent ALK inhibitor produced by Chugai Pharmaceutical Co., a Japanese company (Chuo, Tokyo) of the Hoffmann-La Roche group and approved by FDA and EMA in 2015 and 2017, respectively, for the treatment of advanced ALK-positive NSCLC after crizotinib treatment failure. In 2018 it was also approved as a first-line treatment for the same disease.

Song et al. reported that the compound displays activity toward ALK mutations, including L1196M, F1174L (which can arise de novo after crizotinib treatment), R1275Q, and C1156Y, all conferring resistance to crizotinib [188]. Regarding NB, alectinib has antiproliferative effects on NB cell lines bearing wild-type, F1174L- and D1091N-mutated ALKs. It acts by inhibiting ALK-mediated PI3K/AKT/mTOR signaling. The compound potentiates the effect of the anticancer drug doxorubicin and is active in the xenograft NB mouse model [189]. 

The preclinical potential of alectinib in ALK-positive NB has further been investigated by Alam et al. who evaluated the compound’s ability to abrogate the activity of different full-length ALK gain-of-function mutations found in certain NB cases. The study was performed by evaluating neurite outgrowth, cell cycle progression, and induction of apoptosis. The authors tested alectinib on a panel of NB cell lines, including CLB-BAR (MYCN/ALK-amplified), CLB-GE (MYCN/ALK-amplified and ALK-F1174V), CLB-PE (MYCN-amplified), and IMR-32 (MYCN-amplified), using crizotinib as a positive control. The compound is particularly active in inhibiting the proliferation of CLB-BAR and CLB-GE cells, with IC_50_ values of 70.8 and 84.4 nanomolar, respectively. As result, the authors observed a reduction of ALK activity, a decrease in phosphorylation of the downstream signaling targets AKT and ERK1/2, and, also, a reduction in MYCN protein levels [190]. 

Alectinib has been tested in association with the histone deacetylase inhibitor vorinostat, a drug approved for the treatment of resistant forms of cutaneous T-cell lymphoma. The combination has a synergistic antiproliferative effect on NB cell lines harboring the R1275Q mutation of ALK and leads to an increased caspase-dependent apoptosis. Furthermore, it reduces the levels of MYCN and the nuclear factor kappa B, a protein with an important role in NB [191].

The crystal structure of the human ALK in complex with complex tetracyclic (5,6-dihydro-11*H*-benzo[*b*]carbazol-11-one) alectinib (PDB code: 3AOX)[192] confirms its binding into the ATP site in the DFG-in mode. The benzo[*b*]carbazole moiety is located in the flat pocket between the *N*- and *C*-lobes and its carbonyl oxygen is involved in a crucial hydrogen bond in the hinge region. The overall hydrophilic interactions are fundamental to achieving target selectivity [193]. 

#### 4.1.3. Ceritinib 

The substituted diphenylamino-pyrimidine ceritinib (LDK378) (**43** in Figure 6) [194] is a second-generation ALK inhibitor (GI_50_ = 0.15 nM). Crystallographic studies of its complex with ALK (PDB code: 4MKC) [195] show the pyrimidine ring in the hinge region and the chlorine atom participating in a hydrophobic interaction [196]. 

Ceritinib has been approved by FDA and EMA in 2014 and 2017, respectively, as the first-line treatment of NSCLC in ALK fusion-positive patients, such as inhibitors crizotinib and alectinib. In addition to inhibiting ALK, the compound is effective against insulin-like growth factor receptor-1 (IGF-1R), STKK22D, and INSR. Ceritinib is able to overcome both ALK-crizotinib resistance mutations (G1269A, L1196M, I1171T/N, and S1206C/Y) and ALK-alectinib resistance mutations (I1171T/N/S and V1180L) in NSCLC [196].

The compound originally exhibited activity against the ALK mutation F1174L in NB cells [197], but, later, it was found ineffective in cells endowed with such mutation. Debruyne et al. investigated the mechanisms that could cause acquired resistance to ALK inhibitors, including ceritinib, in ALK^F1174L^-driven NB, to find other targets that may be exploited to treat the disease. The authors demonstrated that the activation of the TK AXL, through upregulation of the ERK pathway, is partially responsible for the resistance to the mutation ALK^F1174L^. An additional mechanism could be due to the epithelial-to-mesenchymal transition (EMT) in mutated NB cells. For this reason, associations of ALK and AXL or HSP90 inhibitors could be useful to delay the appearance of the mutation ALK^F1174L^ in NB patients [198]. A recent study demonstrated the activity of ceritinib on a HR-NB 16-months-old patient with metastatic disease and not tolerating the toxicity of conventional chemotherapeutic drugs. The initial biopsy revealed the gain-of-function mutant ALK^I1171T^. Different ALK inhibitors were used and ceritinib showed an 11-fold improved inhibition of ALK^I1171T^ when compared with crizotinib. Thus, its monotherapy was selected to treat this type of childhood tumor, being well-tolerated and resulting in tumor shrinkage [199]. 

#### 4.1.4. Brigatinib

Brigatinib (AP26113) (**44** in Figure 6) is a multikinase inhibitor by Ariad Pharmaceuticals. Similarly to ceritinib, it is a diphenylamino-pyrimidine derivative, but differs from the presence of the dimethylphosphonic group on the terminal phenyl ring in place of the isopropylsulfonyl group and bears also a basic portion on the other substituted phenyl ring. It is active on ALK, including many mutated forms, EGFR and ROS1. It is classified as a second-generation ALK inhibitor. In 2017, the compound was approved both by FDA and EMA to treat NSCLC patients harboring ALK mutation and patients after crizotinib-failed therapies. In 2020, it was approved as a first-line drug for the same disease.

Moreover, brigatinib has been explored in preclinical NB models, including several cell lines, mouse xenografts, and Drosophila melanogaster model systems expressing constitutively active ALK mutations. Brigatinib has been shown to inhibit ALK more effectively than crizotinib [200].

The brigatinib-ALK complex (PDB code: 6MX8) [201] highlights the fitting of the inhibitor in the ATP-binding site through a U-shaped conformation, stabilized by intramolecular interactions established by the peculiar dimethylphosphine oxide-anilino moiety. The pyrimidine ring binds in the adenosine site, while the methoxy group and the aniline are in proximity of the hinge and DFG sites, respectively. Additionally, the substituents on the bisanilinopyrimidine scaffold seem to be responsible for the inhibitory potency [202]. 

#### 4.1.5. Lorlatinib

Lorlatinib (PF-06463922) (**45** in Figure 6) is a third-generation highly potent ALK/ROS1 inhibitor by Pfizer. In an enzymatic assay towards the isolated kinases, the compound showed K_i_ values lower than 0.02 and 0.07 nM for ROS1 and ALK, respectively [203]. In 2018, lorlatinib was approved by both FDA and EMA for the second- or third-line treatment of ALK-positive metastatic NSCLC. It overcomes almost all known ALK resistance mutations observed with other ALK inhibitors, including the ALK-G1202 mutation [180]. Interestingly, Lorlatinib showed IC_50_ values ranging from 2.8 to 26.6 nM in an in vitro assay on nine cell lines expressing different ALK mutations resulting from 20 to 125-fold more active than crizotinib [204]. 

In preclinical NB models, lorlatinib exhibits superior potency toward ALK compared to the other inhibitors. Guan et al. reported that the compound shows activity both in vitro and in vivo NB models harboring ALK mutations, such as a mouse model of HR-NB driven by Th-ALK^F1174L^/MYCN, on which crizotinib resulted inactive [205]. In the same year, Infarinato et al. demonstrated the higher activity of lornatinib compared to crizotinib in both in vitro and in vivo studies. In more detail, the authors found that the compound induces tumor regression in both crizotinib-resistant and crizotinib-sensitive xenograft mouse models of NB as well as in patient-derived xenografts with the crizotinib-resistant F1174L or F1245C mutations [204]. However, in the last few years, resistance to lornatinib has been sometimes observed. Radaelli et al. selected a NB cell line resistant to lornatinib (CLB-GA-LR1000) to perform a whole-exome sequencing and proteomic profiling of these cells and revealed a truncating NF1 mutation and hyperactivation of EGFR and ErbB4 [206].

Lorlatinib is a 12-membered macrocycle bearing several heterocycle rings fused, such as a pyrazole, a phenyl, and a pyridine. From its complex with ALK (PDB code: 4CLI) [207], the pyrazole cyano moiety emerges as a fundamental motif for obtaining selectivity and the aminopyridine binds the hinge region, providing the required hydrogen bonding donor groups [203].

#### 4.1.6. Entrectinib

Entrectinib (RXDX-101) (**46** in Figure 6) by Ignyta, is a substituted 1*H*-indazole derivative that inhibits ALK, tropomyosin receptor kinases (TRKs) A, B, and C, and ROS1, with IC_50_ of 1.7, 0.1,0.1, 0.2, and 1.6 nM, respectively [208]. It received the FDA and EMA approvals in 2019 and 2020, respectively, for the treatment of ROS1-positive NSCLC and NTRK fusion-positive solid tumors. In Europe, it has orphan drug designation for the treatment of NB [209]. Entrectinib has been studied in preclinical models of different solid tumors, where it showed activity toward the ALK-G1202R mutant [208]. 

Iyer et al. evaluated the in vitro effects of entrectinib, either as a single agent or in combination with irinotecan and temozolomide, on SH-SY5Y NB cells transfected with TRKB. The authors reported that the compound used alone inhibits the growth of these cells with a potency in the nanomolar range, and the combination has a synergistic effect. Entrectinib resulted to be active also in NB xenografts, in mono- and polytherapy [210]. 

In the same year, Aveic et al. tested in vitro entrectinib for its antiproliferative activity on NB cell lines with different statuses of ALK gene (wild-type, mutated, or amplified). They found that the compound inhibits the growth of cells with amplified ALK, while it is less active on cells harboring the mutations *ALK*^F1174L^, and *ALK*^R1275Q^) or wild-type ALK. The authors also reported that entrectinib induced autophagy and this mechanism could be responsible for drug resistance. Inhibition of autophagy by chloroquine improved cell susceptibility to the compound [211]. 

MacFarland et al. obtained further insight into the mechanism of entrectinib resistance, shown by some NB cell lines. The cells were established from five NB xenografts initially sensitive to entrectinib therapy. No TRKB gene mutation was observed in resistant cell lines, while ERK/MAPK upregulation was observed in all resistant cell lines. The author observed increased IGF1R signaling in a clone and concluded that entrectinib resistance could depend on different mechanisms, including activation of ERK/MAPK, increased IGF1R signaling, and downregulation of PTEN signaling. This information may be useful in the planning of phase II/III trial on the drug for the treatment of NB [212].

#### 4.1.7. TAE684 

TAE684 (NVP-TAE684) (**47** in Figure 6) is one of the first ALK-specific inhibitors identified to target the ATP-binding site of ALK. Chemically, it is characterized by a substituted 5-chloro-2,4-diphenyl-chloropyrimidine scaffold., analogously to its derivatives ceritinib and brigatinib. It reduced cell proliferation in ALK-positive anaplastic large cell lymphoma (ALCL), which is frequently characterized by the fusion protein nucleophosmin (NPM)-ALK [213]. 

Regairaz et al. showed that TAE684 is active in preclinical models of NB. The compound has antiproliferative activity on NB cell lines, especially those with ALK mutation. It inhibits ALK phosphorylation in xenografts models harboring the ALK mutation F1174I and R1275Q but possesses antitumor activity only against the R1275Q xenograft [214]. 

Najem et al. reported that the association of TAE684 and the SMAC (second mitochondria-derived activator of caspase) mimetic derivative LCL161 has synergistic activity in NB cells characterized by ALK mutation F1174L. SMAC mimetics are small molecules that can induce apoptotic cancer cell death and block pro-survival signaling in cancer cells [215]. 

Powell et al. synthesized the first examples of derivatives that induce the degradation of ALK in ALCL, NSCLC, and NB cell lines. The NB cell lines used in this study had either the ALK mutation F1174L or R1275Q. The compounds derived from the conjugation of TAE684 or ceritinib with pomalidomide (a molecule that targets the protein cereblon) are able to bind ALK with IC_50_ values similar to those of their parental inhibitors. Cereblon in turn recruits the E3 ubiquitin ligase, which belongs to a protein complex that ubiquitinates and consequently degrades different proteins, including ALK. The most active compound bears the same core of TAE684 linked through an ethyleneglycol spacer to an isoindoline-1,3-dione scaffold (**48** in Figure 6) on NB cells is that derived from the fusion of ceritinib and pomalidomide [216]. This study reports an innovative approach for the inhibition of ALK mutated NB cell lines.

ALK-TAE684 cocrystal structure (PDB code: 2XB7) [217] reveals the establishment of hydrogen bonds between the inhibitor pyrimidine and amino nitrogens and the hinge region, and these are the only direct polar contacts in the complex. The isopropylsulfonyl phenyl ring fits into a cavity and establishes hydrophobic interactions, such as the piperidinyl-1-methylpiperazine group [218].

#### 4.1.8. Other Studies on ALK Inhibitors in NB Models

Recently, Emdal et al. performed quantitative mass spectrometry-based proteomics on NB cells treated with one of the four most potent ALK inhibitors (crizotinib, ceritinib, lorlatinib, and TAE684). They identified the signaling adaptor protein insulin receptor substrate 2 (IRS2) as an important ALK target. In fact, the treatment with the TK inhibitors decreases the recruitment of IRS2 to ALK and reduces the tyrosine phosphorylation and activation of IRS2. The authors also reported the involvement of IRS2 in the signaling pathway PI3K-AKT-FoxO3 in NB cells. Moreover, short interfering RNA (siRNA)-mediated depletion of ALK or IRS2 inhibits the transcription factor FoxO3. This leads to a reduction of the viability of three ALK-driven NB cell lines (NB1: ALKAmp, SH-SY5Y: ALKF1174L, and CLBGA: ALKR1275Q) [219]. 

Watts et al. reported that the concomitant inhibition of ALK and BRD4 is a potential therapeutic strategy for the treatment of HR-NB patients [220]. BRD4 is a protein involved in the promotion of gene transcription and has emerged as an essential transcriptional co-regulator of MYCN. Inhibition of BDR4 resulted to be an effective therapeutic approach to target dysregulated MYCN in NB [169]. Starting from a known BRD4 inhibitor, the authors designed and synthesized a dual ALK-BRD4 inhibitor. The most active compound contains a 7,8-dihydropteridin-6(5*H*)-one core (**49** in Figure 6), and possesses an IC_50_ value of 17 nM on ALK F1174L and a K_d_ value of 44 nM on BRD4, with a good selectivity on many other kinases in enzymatic assays. In cells, **49** shows a potency of 470 nM against ALK and 260 nM against BRD4 [220]. 

Later, Munira et al. get further insights into the activity of the most potent ALK inhibitors crizotinib, ceritinib, and TAE684 on NB cells. The authors choose the SH-SY5Y NB cell line since the ALK expression is higher in this line than in other NB cells and reported that ALK inhibitors reduced the proliferation of SH-SY5Y NB cells in a concentration-dependent way. The activity of such compounds led to misorientation of spindles, misalignment of chromosomes, and reduction in autophosphorylation of Alk, and these effects caused an M phase delay [221]. 

### 4.2. TRK Inhibitors

The TRK family is constituted by RTKs activated by the neurotrophin family. In detail, it includes three isoforms: TRK-A (NTRK1), TRK-B (NTRK2), and TRK-C (NTRK3), and their primary ligands are nerve growth factor (NGF), brain-derived neurotrophic factor (BDNF), and neurotrophin-3 (NT3), respectively. The binding of neurotrophins to TRKs induces receptor dimerization, phosphorylation, and activation of the downstream signaling cascades via AKT, MAPK, and PLC. Among other functions, they are involved in tumor development [222]. In NB, TRK-A expression is associated with a positive outcome, since it mediates cell differentiation, growth regulation, and reduction of angiogenesis in response to NGF [223], while the TRK-B/BNDF pathway is frequently expressed in the most severe NB variants, in which it promotes cell survival and reduced sensitivity to cytotoxic anticancer agents [224]. 

Several TRK inhibitors have been developed and studied for the treatment of many cancers [225], including NB. Among TRK inhibitors tested against NB, entrectinib has been already reported in the section of ALK inhibitors.

#### 4.2.1. AZ-23

**Figure 7:**
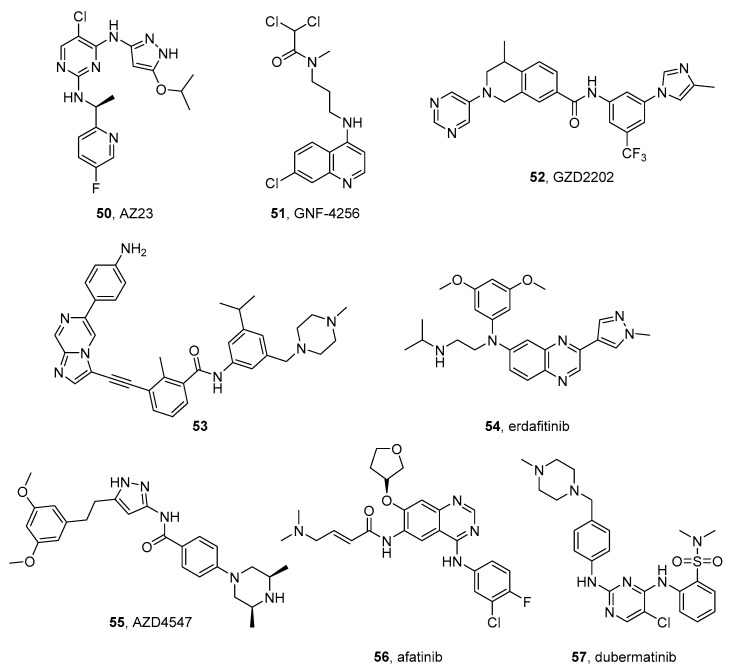
Other RTK inhibitors.

Some years ago, Thress and colleagues identified a nanomolar and selective TRK inhibitor, AZ-23 (**50** in Figure 7), active both in vitro and in vivo assays. Interestingly, the compound inhibits tumor growth in a TRK-expressing xenograft model derived from SK-NSH NB cells. In this study, the authors demonstrated the potential efficacy of TRK inhibition in a NB setting [226]. 

Human TRK-A in complex with the inhibitor AZ-23 (PDB code: 4AOJ) [227] shows that the enzyme adopts an inactive conformation, the pyrazole of the inhibitor forms hydrogen bonds to its backbone, while the relevant contacts are observed for the fluoropyridine ring. Moreover, the pyrimidine N1 atom is involved in a water bridge in a solvent-exposed region of the ATP-binding site [228]. 

#### 4.2.2. GNF-4256

GNF-4256 (**51** in Figure 7) is a quinoline derivative acting as a selective pan-TRK inhibitor by Novartis. It inhibits TRK-A, -B, and -C with nanomolar IC_50_ values. Croucher et al. reported the in vitro and in vivo activity of this compound on NB cells and models, alone or in combination with irinotecan and temozolomide. GNF-4256 inhibits the phosphorylation of TRK-B in a dose-dependent manner and has antiproliferative activity on TRK-B-expressing human NB cells (SY5Y-TRK-B), with an IC_50_ value of 50 nM. This growth inhibition is due to TRK-B inhibition since the compound does not have any effect on SY5Y TRK-null cells. It also inhibits the growth of NB xenograft mice models at doses of 40 or 100 mg/kg twice a day, without evident signs of toxicity. GNF-4256 increases irinotecan and temozolomide activity in these models, probably reducing the results of BDNF/TRK-B overexpression, including cell motility and invasion, angiogenesis, and metastasis formation [229]. 

#### 4.2.3. GZD2202

Recently, Zou et al. reported the novel TRK-B inhibitor, GZD2202 (**52** in Figure 7), that shows a moderate selectivity among TRK-B/C and TRK-A. It inhibits the brain-derived neurotrophic factor (BDNF), which is mediated by TRK-B, and reduces proliferation, migration, and invasion in SH-SY5Y-TrkB NB cells. The inhibitor also has antitumor activity in a SH-SY5Y-TRK-B xenograft model [230]. 

Molecular docking simulations suggest GZD2202 binds TRK-B as a type-II inhibitor. Its pyrimidine moiety establishes an essential hydrogen bond in the hinge region and the trifluoromethyl group interacts with the DGF motif in the activation loop [230].

#### 4.2.4. Other Compounds

A series of 3-(imidazo[1,2-*a*]pyrazin-3-ylethynyl)-2-methylbenzamides resulted active as pan-TRK inhibitors. The most active compound has a 3,4-dihydro-isoquinoline core (**53** in Figure 7) and inhibits TRK-A, B, and C with IC_50_ values of 2.65, 10.47, and 2.95 nM, respectively. Moreover, this derivative has antiproliferative effects (IC_50_ = 58 nM) on SH-SY5Y cells overexpressing TRK-B. In these cells, it suppresses migration and invasion. Pharmacokinetics studies suggest that the compound has a periferically restricted activity, avoiding the potential neurotoxic issue of a pan-Trk inhibitor [231]. 

Very recently, the activity of a new dual inhibitor of TRKs and AURKA, named TAS-119, whose structure is not disclosed, has been reported. The compound inhibits TRK-A, B, and C, with IC_50_ values of 1.46, 1.53, and 1.47 nM, respectively, and AURKA with an IC_50_ value of 1.04 nM, with a good selectivity on a panel of other kinases. TAS-119 induces N-Myc degradation and inhibits downstream targets in MYCN-amplified NB cell lines [232].

### 4.3. FGFR Inhibitors

Fibroblast growth factor receptors (FGFRs) constitute a family of transmembrane TKs frequently involved in malignancies. Among FGFR inhibitors tested in cancer [233], only two compounds have been tested on NB and are herein reported.

#### 4.3.1. Erdafitinib

Erdafitinib (JNJ-42756493) (**54** in Figure 7), by Janssen, is a potent FGFR inhibitor with quinoxaline structure. It inhibits FGFR1/2/3/4 with IC_50_ values of 1.2, 2.5, 3.0, and 5.7 nM, respectively, and has been recently approved by FDA for the treatment of advanced or metastatic urothelial carcinoma and is in clinical trials for many other cancers [234]. As reported above (see the paragraph on PI3K inhibitors), the association of erdafitinib with alpelisib inhibits NB cell growth and can be used in combination with specific cytotoxic agents [138]. 

The complex of erdafitinib with FGFR1 (PDB code: 5EW8) [235], the inhibitor is located into the enzyme ATP-binding cleft, and the activation loop exhibits a DFG-in conformation. Crucial hydrophilic interactions are observed for the quinoxaline core, the methoxyl oxygen atom, and the amine side chain which extends into the region usually occupied by the ATP first phosphate group. Moreover, the terminal isopropyl group establishes Van der Waals interactions in a shallow pocket named the “pit” region [236,237]. 

#### 4.3.2. AZD4547

AZD4547 (**55** in Figure 7) inhibits FGFR members with IC_50_ values in the nanomolar range (0.2, 2.5, 1.8, and 165 nM for FGFR1, FGFR2, FGFR3, and FGFR4, respectively). This compound is being tested in many clinical trials as an anticancer agent.

Analogously to erdafitinib, AZD4547 has been tested in association with PI3K inhibitors (dactolisib and BKM120). Kostopoulou at al. assessed the activity of the compound, either alone or in association, on 5 NB cell lines (SK-N-AS, SK-N-BE(2)-C, SK-N-DZ, SK-N-FI, and SK-N-SH). When in monotherapy, the compound generally reduces cell viability and proliferation, while the association with dactolisib leads to increased antiproliferative activity on all the tested cell lines [135]. 

Recently, AZD4547 has been tested on several aggressive pediatric tumor cells, including NB, rhabdomyosarcoma, and Ewing sarcoma. AZD4547 treatment causes cell confluence, increases apoptosis, and inhibits cell migration. The activity of this compound is probably due to its effects on the Ras/MAPK and JAK/STAT pathways [238].

The structure of FGFR1 in complex with AZD4547 (PDB ID: 4V05) [239] confirms a type-I inhibitor binding mode. The inhibitor is in the ATP-binding cleft of FGFR1 and its pyrazole amide moiety forms three hydrogen bonds with the hinge region. The dimethoxyphenyl group fits into the hydrophobic pocket and one of the methoxy oxygen atoms is also involved in a hydrogen bond with the DFG backbone [240]. 

### 4.4. EGFR Inhibitors

The ErbB family of proteins is a group of RTKs that consists of epidermal growth factor receptor (EGFR) (ErbB1 or HER1), ErbB2 (HER2 or Neu), ErbB3 (HER3), and ErbB4 (HER4). These enzymes are involved in the progression of various types of cancers [241] including NB, where EGFR is expressed in high levels.

#### Afatinib

Afatinib (BIBW 2992) (**56** in Figure 7) is a potent EGFR inhibitor with an IC_50_ value of 0.5 nM in enzymatic assays. It is one of the few examples of irreversible kinase inhibitors. The acrylamide portion of the compound binds the cysteine SH group in the enzyme catalytic site through a Michael addition reaction. 

This inhibitor has been approved for the treatment of NSCLC, especially in the presence of EGFR mutations [242]. The cytotoxicity of afatinib was evaluated on six NB cell lines (IMR-32, NGP, NB-19, SK-N-AS, SH-SY5Y, and LA-N-6). The compound has antiproliferative activity on these cells, which induces apoptosis, inhibits EGFR and its downstream pathway PI3K/AKT/mTOR. The association of afatinib with doxorubicin improves the cytotoxicity also on the chemoresistant LA-N-6 NB cell line. Furthermore, afatinib shows anticancer activity in an orthotopic xenograft NB mouse model [243].

The enzyme-ligand cocrystal structure (PDB code: 4G5J) [244] reveals a long hydrogen bond between the hinge region and the quinazoline core and the covalent nature of the afatinib bonding to Cys797 at the edge of the active site [245].

### 4.5. AXL Inhibitors

Besides the well-known prognostic factors for NB, such as ALK and MYCN, functional analyses that followed the whole-genome sequencing confirmed the relevance of additional targets that might lead to the finding of a new treatment for NB [246] or provide new insights about known targets [247]. 

Tyro3, AXL, and Mer (TAM) constitute a family of RTKs that has been proposed to be of particular relevance in conferring survival advantages to cancer cells [248]. These kinases are important tumor propagators and play roles in cell migration, adhesion, and response to drugs. Recently, their involvement in NB pathology has been widely reported. Li et al. demonstrated that Mer and AXL were co-expressed in NB cells and patients and could be involved in metastasis formation. The activation of these enzymes increases the phosphorylation levels of oncogenic pathways of the kinases ERK1/2, AKT, and FAK, while their inhibition causes apoptosis, reduces cell migration, and increases the sensibility of NB cells to vincristine and cisplatin [249]. 

The pyrimidino derivative dubermatinib (TP-0903) (**57** in Figure 7) has a scaffold similar to that of ceritinib, is an orally available multitargeted kinase inhibitor and one of its main targets is AXL [250]. Aveic and et al. tested this inhibitor on several NB cell lines and found a remarkable pharmacological efficacy at sub-micromolar concentrations. The compound impairs colony and neurosphere formation, migration, cell cycle advancement and inhibits NB cells intravasation in vitro and in vivo. Dubermatinib is active not only as a mono-therapy but also in combination with conventional chemotherapic drugs (such as ATRA, cisplatin, and VP16) in different types of NB cells [251]. 

### 4.6. FAK Inhibitors

**Figure 8:**
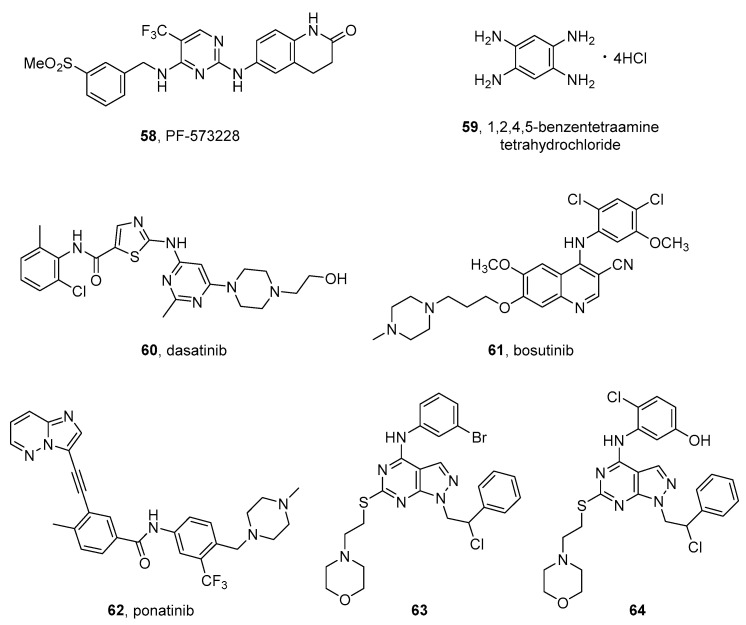
nRTK inhibitors.

Focal adhesion kinase (FAK) is a non-receptor tyrosine kinase (nRTK) that plays important roles in many tumors [252], including NB [253]. It activates pro-survival pathways such as Src, Ras-ERK, and PI3K-AKT, while it inhibits the tumor suppressor protein p53 [254]. FAK overexpression has been connected to aggressive cancer phenotypes [255]. Beierle et al. demonstrated that FAK inhibition decreased survival, migration, invasion, and metastases on NB cells with MYCN amplification [256]. Recently, the same group [257] tested two known FAK inhibitors, the 3,4-dihydroquinolin-2(1*H*)-one-containing PF-573228 (**58** in Figure 8) [258] and 1,2,4,5-benzentetraamine tetrahydrochloride (**59** in Figure 8) [259], which inhibit auto-phosphorylation of FAK at Y3977 with low nanomolar potencies in enzymatic assays, on two MYCN-amplified patient-derived NB xenografts, namely COA3 and COA6. The two compounds inhibit migration and invasion of NB cells, decrease tumorspheres formation and reduce established stem cell markers, determined by immunoblotting and quantitative real-time PCR. The authors demonstrated that FAK inhibition decreases many features of the malignant NB phenotype, among which the cancer stem cell-like, typical characteristics of HR NB models [257]. 

### 4.7. Dual Src/Bcr-Abl Inhibitors

Src is a cytoplasmic TK belonging to the Src family kinase (SFK). Together with other members of this family, Src is hyperactivated and/or overexpressed in a variety of hematological and solid tumors, including NB [260]. Another cytoplasmic TK, Brc-Abl, derives from a chromosomal translocation that leads to the formation of the Philadelphia chromosome, codifying for this constitutively activated kinase. Bcr-Abl is present in the majority of cases of chronic myeloid leukemia (CML) [261]. Src and Bcr-Abl share a similar structure and compounds identified as Src inhibitors frequently resulted also Bcr-Abl inhibitors. However, the majority of dual Scr/Bcr-Abl inhibitors are also active on many kinases and can be defined as multitargeted.

#### 4.7.1. Dasatinib

The most studied of dual inhibitors is dasatinib (BMS-354825) (**60** in Figure 8), by Bristol-Myers Squibb, approved in 2006 as a second-line treatment for CML in patient’s refractory to previous treatments, and, more recently, as a first-line drug for this cancer. Moreover, the compound is being tested in a very huge number of trials for the treatment of solid and hematological malignancies, including NB. In 2009, Vitali et al. reported a potent activity of Dasatinib on HTLA-230 and SY5Y NB cell lines, which is due to Src, c-Kit, and AKT inhibition. In orthotopic xenograft models, the anti-tumor effect of dasatinib was partial [262]. The difference between the results obtained in vitro and in vivo with dasatinib could be explained by the inhibition of the immune system in vivo mediated by Src inhibition [263]. 

Dasatinib has an aminopyridine core linking a hydroxyethyl-piperazine and a 2-amino-1,3-thiazolecarboxamide groups in C6 and C4, respectively. According to the X-ray diffraction studies on the Abl-dasatinib cocrystal (PDB code: 2GQG) [264], the inhibitor sits in the ATP-binding site: the aminothiazole ring binds the ATP-adenine site through hydrogen bonds and, also, both thiazole and pyrimidine rings interact with carbonyl oxygens belongings to hinge region residues. The pyrimidine ring in the hydrophobic cleft establishes Van der Waals contacts, while the substituted phenyl ring fits in a hydrophobic pocket. The polar piperazine moiety is located in the hinge region surface-exposed portion through Van der Waals interactions, while the terminal hydroxyethyl group is highly flexible, forming hydrogen bonds or pointing in the solvent-exposed opposite direction [265].

#### 4.7.2. Bosutinib

Bosutinib (**61** in Figure 8), by Pfizer, is a dual Src/Bcr-Abl inhibitor approved in 2017 for the treatment of patients with newly-diagnosed chronic phase CML. Like dasatinib, it also inhibits other kinases. Bieerkehazhi et al. demonstrated that bosutinib has antiproliferative effects on NB cells, by reducing the activity of Src/Abl, PI3K/AKT/mTOR, MAPK/ERK, and JAK/STAT3 pathways. It shows a synergistic effect in combination with doxorubicin and etoposide and possesses anticancer activity in an orthotopic xenograft model [266].

The structural analysis of Bosutinib bound to Abl kinase domain (PDB code: 3UE4) [267] reveals the peculiar feature of the binding mode of this class of inhibitors, consisting of the hydrogen bonding network established by the quinoline N1 nitrogen and the hinge region backbone. Furthermore, the aniline ring sits in a hydrophobic pocket projected into the ATP-binding site, while the *N*-propoxy-*N*-methylpiperazine group extends out of the site, generating Van der Waals contacts with the kinase hinge region [268].

#### 4.7.3. Ponatinib

Ponatinib (**62** in Figure 8), by Ariad Pharmaceuticals, is a third-line drug for the treatment of CML, especially for the forms expressing the gatekeeper mutation T315I, which is resistant to the first and the second line drugs imatinib, nilotinib, dasatinib, and bosutinib. Ponatinib was first identified as a pan-Bcr-Abl and Src inhibitor. Further studies have demonstrated that it is a multitargeted inhibitor active on FGFRs, RET, AKT, ERK1/2, KIT, MEKK2, and other kinases.

Although initial side effects issues were observed, ponatinib received full approval by FDA in 2016 to treat CML patients that have no other therapy options, such as patients with the T315I mutation. Regarding NB, many of the kinases targeted by ponatinib, including FGFR1-4, PDGFR, Src, RET, KIT, FLT3, and VEGFR1 are involved in this disease [28]. 

Whittle et al. tested ponatinib on many NB cell lines (CHP-134, CHP-212, NGP, LAN-5, SH-EP, SK-N-AS, SK-N-BE, and SK-N-SH), showing IC_50_ values in the range 0.9–9.1 µM. It also decreases cell viability and migration and reduces tumor growth and vascularization of the tumor mass in an orthotopic xenograft NB model in mice [269]. Successively, other groups reported similar results on ponatinib activity against NB both in vitro and in vivo. 

Singh et al. examined the activity of Bcr-Abl inhibitors as antiproliferative agents on NB cells. Ponatinib resulted to be the most active compound and, probably, by reducing IGF-1R and Src activity, inhibits cell migration and induces apoptosis [270]. Li et al. confirmed that ponatinib inhibits proliferation and induces apoptosis in NB cells in a dose-dependent manner. Ponatinib also possesses a synergistic effect when is used in combination with doxorubicin in NB cells and inhibits tumor growth in an orthotopic xenograft model in mice [271].

Sidarovich et al. screened a library of 349 anticancer agents, which were tested on the three NB cell lines: CHP-134, IMR-32, and SK-N-BE. Ponatinib emerged as the most active compound both in vitro and in vivo. The authors underlined that ponatinib could be used at a reduced dose (15 mg/day instead of the usual dosage of 45 mg/day), still maintaining its therapeutic activity while decreasing the vascular adverse side effects [272]. 

Very recently, ponatinib was confirmed to be active in vitro on SK-N-BE(2), SH-SY5Y, and IMR-32 human NB cell lines and in vivo in zebrafish and mouse models. Moreover, its combination with chloroquine reduces lysosomal metabolism and autophagic flux, with an increased activity also in in vivo models [273]. 

X-ray data of Ponatinib in complex with the mutated Abl^T315I^ (PDB code: 3IK3) [274] shows the fitting of the imidazo[1,2-*b*]pyridazine core into the adenine pocket of Abl. The tolyl group occupies the hydrophobic pocket behind the gatekeeper residue and the trifluoromethylphenyl group binds tightly to the pocket induced by the DFG-out conformation of the protein. In addition, the ethynyl linkage interacts with the side chain of I315, allowing inhibition of the T315I mutant. The latter structural moiety also acts as an inflexible connector that stabilizes and enforces the binding affinity [275]. 

#### 4.7.4. Other Compounds

Several members of an in-depth investigated family of pyrazolo[3,4-*d*]pyrimidines by Schenone’s and Botta’s research groups developed as Src inhibitors [276,277,278] resulted active in preclinical models of NB [279,280,281]. In particular, the 3-bromoaniline-bearing compound Si306 (**63** in Figure 8) shows a potent antiproliferative activity on NB cell lines and is active in a xenograft model of SH-SY5Y cells [279]. As most of the kinase inhibitors, also this pyrazolo[3,4-*d*]pyrimidines class suffers from low water solubility, thus, the same authors formulated drug-loaded human serum albumin (HSA) nanoparticles, to improve pharmacokinetic properties. The nanoparticles were tested for their antiproliferative activity against NB SH-SY5Y cell line and some of these formulations possess a profitable balance of stability, size, and biological activity comparable to that of the free compounds in cell-based assays [280]. Then, the authors performed an optimization study which led to the identification of a new series of derivatives, whose representative compound is **64** (in Figure 8) endowed with nanomolar K_i_ values against c-Src, interesting antiproliferative activity on SH-SY5Y cells, and a suitable ADME profile [281].

## 5. Multitargeted Inhibitors

Many kinase inhibitors act on a certain number of different kinases and are defined as multitargeted. Several compounds of such a type entered the clinical use since frequently more than one kinase is hyperactivated or overexpressed in malignancies. 

### 5.1. Sorafenib

**Figure 9:**
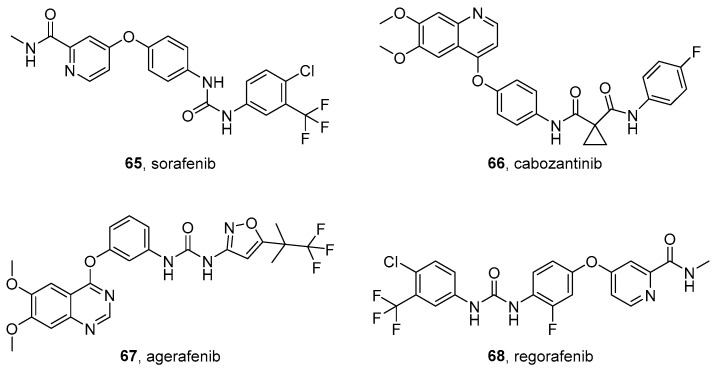
Multikinase inhibitors.

Sorafenib (**65** in Figure 9) is an orally available inhibitor of multiple kinases, including C-RAF, B-RAF, c-KIT, FLT3, platelet-derived growth factor receptor (PDGFR) α and ß, and vascular endothelial growth factor receptor (VEGFR) 1, 2, and 3 [282]. Sorafenib has been reported to increase apoptosis in human NB cell lines and downregulate the ERK, AKT, RAF-MEK, and JAK2-STAT3 survival pathways. The compound has been evaluated in pediatric patients with solid tumors or leukemia in two phase I studies in combination with other anticancer agents and, thereafter, as single-agent therapy. Okada et al. reported the data obtained after treatment with sorafenib in four patients affected by treatment-refractory NB. Sorafenib showed a transient activity in all four patients without adverse effects, but after a short period, the disease restarted. Probably, the compound could be active in earlier stages of NB [283]. 

The crystal structure data of the isolated B-RAF kinase domain with Sorafenib (PDB code: 1UWH) [284] shows the distal pyrimidine ring of the inhibitor into the ATP adenine binding pocket and the lipophilic trifluoromethyl phenyl group inserted into a hydrophobic pocket. Although hydrophobic interactions are predominant, polar interactions also contribute to binding. In particular, the urea group of the inhibitor forms two hydrogen bonds with the protein, one between the amide nitrogen and a catalytic residue and the second one between the carbonyl oxygen and the DFG motif [285].

### 5.2. Cabozantinib

Cabozantinib (**66** in Figure 9), by Exelixis. Inc., is a multitargeted inhibitor of RTKs, including MET, RET, AXL, VEGFR2, FLT3, and c-KIT. It has been approved by FDA and EMA for the treatment of medullary thyroid cancer and renal cell cancer [286]. 

Zhang et al. reported that cabozantinib, used alone or in combination with 13-cis-retinoic acid, topotecan, and temozolomide has antiproliferative effects on many NB cells, with IC_50_ values in the range from 1.6 to 16.2 μM when used alone. The compound shows a synergic effect with other drugs. This inhibitor decreases RET phosphorylation in all cell lines and reduces ERK phosphorylation in more sensitive NB cells. In orthotopic xenograft models in SCID mice, cabozantinib reduced tumor growth in a significant way [287]. The importance of the simultaneous inhibition of many kinases is demonstrated by the study of Daudigeos-Dubus et al. that observed that cabozantinib orally administered per gavage at 30 and 60 mg/kg/day inhibits tumor growth of orthotopic adrenal IGR-N91-Luc and metastatic IMR-32-Luc NB models in a dose-dependent manner, while axitinib, a potent VEGFR1-3 inhibitor, leads to increased metastasis formation especially in NB models using severely immunocompromized BalbC RAGγC mice. The antitumor activity of cabozantinib is associated with decreased vascularization, inhibition of p-SRC, and induction of apoptotic cell death [288]. 

The better activity profile of cabozantinib compared to that of axitinib could be explained since VEGFR inhibitors can increase Met expression in many tumors [289], and, in turn, Met is overexpressed in NB cells and furnishes an alternative pathway to NB cells to survive [290].

More recently, Perisa et al. reported the results of a clinical trial performed on four children with relapsed metastatic NB treated with cabozantinib. All four patients had extended disease control, with two complete responses for more than 12 months, and two stable diseases for over 6 months. Dose reduction was needed in two patients to reduce predictable toxicities [291]. 

As regards its chemical structure, cabozantinib is a substituted malonamide endowed with a quinoline ring. 

### 5.3. Agerafenib 

The *N*,*N*’-disubstituted urea Agerafenib (RXDX-105, CEP-32496) (**67** in Figure 9) is a potent inhibitor of c-RAF and its mutants (in particular BRAF (V600E/wild-type) Abl-1, c-Kit, Ret, PDGFRβ, and VEGFR2, with IC_50_ values in the nanomolar range [292]. 

Li et al. reported that high expression of RAF is correlated with HR-NB and poor survival. Agerafenib, by inhibiting RAF, reduces the activation of the ERK/MAPK pathway and has antiproliferative and proapoptotic effects on NB cells. Moreover, the compound has anticancer activity in vivo, with an acceptable toxicity profile [293]. In the same year, Flynn et al. confirmed the activity of agerafenib on NB models and reported that it has antiproliferative and apoptotic effects on 11 NB cell lines. Its activity is due to the inhibition of the phosphorylation of Ret, MeK, and Erk. The compound also inhibits angiogenesis and reduced tumor size in SK-N-AS and SK-N-SH xenograft models and demonstrates enhanced efficacy when combined with 13-cis-retinoic, which is a vitamin A analog used for maintenance therapy in HR-NB patients [294]. 

### 5.4. Regorafenib

Regorafenib (**68** in Figure 9), developed by Bayer, is a diphenylurea multi-kinase inhibitor that is active on angiogenic (VEGFR1-3, TIE2), stromal (PDGFR-β, FGFR), and oncogenic RTKs (KIT, RET, and RAF) [295]. In 2012 it was approved for the treatment of metastatic colorectal cancer, in 2013 for gastrointestinal stromal tumors in patients previously treated with imatinib and sunitinib, and later, in 2018, for the treatment of hepatocellular carcinoma in patients previously treated with sorafenib. RET, which is one of the regorafenib targets, is frequently expressed in NB, and correlates with poor prognosis. Chen et al. reported that regorafenib inhibits glial cell-derived neurotrophic factor (GDNF)-induced RET signaling in NB cells and has antiproliferative effects both in vitro and in vivo in NB models [296]. 

Very recently, Subramonian et al. confirmed that regorafenib leads to a reduced NB cell viability and confluence, induces apoptosis and cell cycle arrest. The compound is also effective against NB tumors in vivo, and its association with 13-cis-retinoic acid demonstrates enhanced efficacy compared with regorafenib alone [297]. 

## 6. Conclusions

NB is the most common extracranial solid tumor occurring in children. Its high heterogeneity is one of the major causes of the limited efficacy of different targeted therapies and poor outcomes [298]. Many kinase inhibitors are being tested for the treatment of NB. So far, none of these compounds has been approved, alone or in combination, to treat NB, but many inhibitors are currently in clinical trials, as reported in Table 1. Even if several compounds show good activity in vivo, probably the potential future use of this type of compounds will be in combination with other anticancer agents, because of the complexity and the severity of the aggressive forms of this disease.

An interesting therapy could be constituted by the combination of kinase inhibitors with autophagy inhibitors, such as chloroquine, reported by Aveic et al. [299].

In a recent review, Moreno et al. reported the results of the Second Neuroblastoma Drug Development Strategy forum from Innovative Therapies for Children with Cancer and International Society of Paediatric Oncology Europe Neuroblastoma [300]. Among different kinases, ALK and CDK represent the most promising targets for NB therapy. Inhibitors of these enzymes are endowed with different scaffolds and decorations. A pyrimidine ring is often present in these derivatives, but the complexity of the structures makes the identification of the most promising cores very hard. On the other hand, this variability increases the chances of success in the approval of a new drug since different structures are developed and studied in clinical phases at the same time. Anyway, the challenge to fight this disease must be continued, and probably the combination of agents acting with different mechanisms will lead to encouraging results.

## Figures and Tables

**Table 1:** Kinase inhibitors in clinical trials for NB treatment.

Compound	Pharmaceutical Company	Targeted Kinase	NCT Number	Drug Combined with	Phase	Status
Ribociclib, **2**	Novartis	CDK4/6	NCT01747876	-	1	terminated, has results
			NCT02780128	-	1	recruiting
			NCT03434262	trametinib	1	recruiting
Palbociclib, **3**	Pfizer	CDK4/6	NCT03526250		2	recruiting
			NCT03709680	temozolomide	1	recruiting
Alisertib, **8**	Takeda	AURKA	NCT01601535	iritonotecan temozolomide	1–2	completed
			NCT02444884	-	1	completed
			NCT01154816	-	2	completed
9-ING-41, **18**	Actuate Therapeutics	GSK-3β	NCT04239092	alone or with irinotecan	1	recruiting
SF1126, **30**	SignalRx Pharmaceuticals	PI3K	NCT02337309	-	1	terminated
Rapamycin, **33**		mTOR	NCT01467986	dasatinib irinotecan temozolomide	2	completed
			NCT01331135	-	1	completed
			NCT02574728	celecoxib etoposide cyclophosphamide	2	recruiting
Temsirolimus, **35**		mTOR	NCT01767194	dinutuximab irinotecan sargramostim temozolomide	2	active, not recruiting, has results
			NCT00808899	irinotecan cyclophosphamide doxorubicin etoposide cisplatin topotecan 13-cis-retinoic acid radiation	2	terminated, has results
			NCT01204450	valproic acid	1	terminated
Trametinib, **37**	Novartis	MEK	NCT03434262	gemcitabine ribociclib sonidegib	1	recruiting
			NCT02124772	dabrafenib	1–2	completed
Crizotinib, **41**	Pfizer	ALK	NCT03126916	different anticancer agents	3	recruiting
			NCT01606878	different anticancer agents	1	completed
			NCT00939770	-	1–2	completed, has results
			NCT03107988	lorlatinib cyclophosphamide topotecan	1	recruiting
			NCT01121588	-	1	active not recruiting
Ceritinib, **43**	Novartis		NCT02780128	-	1	recruiting
			NCT02559778	dasatinib sorafenib vorinostat difluoromethylornithine	2	recruiting
			NCT01742286	-	1	completed
Lorlatinib, **45**	Pfizer	ALK	NCT04753658	-	1?	recruiting
			NCT03107988	cyclophosphamide topotecan	1	recruiting
Entrectinib, **46**	Ignyta	ALK	NCT02650401	-	1–2	recruiting
Erdafitinib, **54**	Janssen	FGFR	NCT03210714	-	2	recruiting
			NCT03155620	different anticancer agents	2	recruiting
Dasatinib, **60**	Bristol-Myers Squibb	Src, multitarget	NCT01467986	rapamycin irinotecan temozolomide	2	completed
			NCT02559778	ceritinib sorafenib	2	recruiting
			NCT00788125	carboplatin etoposide ifosfamide	1–2	active, not recruiting
Sorafenib, **65**	Bayer-Onyx	multitarget	NCT02298348	cyclophospamide topotecan	1	recruiting
			NCT02559778	ceritinib dasatinib vorinostat	2	recruiting
			NCT01518413	rinotecan	1	completed
Regorafenib, **68**	Bayer	multitarget	NCT02085148	irinotecan vincristine	1	active, not recruiting
